# Satellite Microwave Radiometry for the Observation of Land Surfaces: A General Review

**DOI:** 10.3390/s26051638

**Published:** 2026-03-05

**Authors:** Cristina Vittucci, Matteo Picchiani

**Affiliations:** 1 Department of Civil Engineering and Computer Science Engineering (DICII), Tor Vergata University of Rome, Via del Politecnico 1, 00133 Rome, Italy; cristina.vittucci@uniroma2.it; 2 Italian Space Agency, Earth Observation Office, Via del Politecnico snc, 00133 Rome, Italy

**Keywords:** microwave radiometry, passive microwave satellite missions, radiative transfer models, vegetation optical depth, snow properties, soil moisture

## Abstract

The development of passive microwave sensors traces back to Robert Dicke’s pioneering experiments in the 1940s. Since then, microwave radiometry has evolved into a key tool for Earth observation, strengthened by data from multiple satellite missions operating across different wavelengths. This paper reviews the state of the art in microwave radiometry for monitoring land surfaces. After introducing the theoretical foundations underpinning current missions, we present an overview of major satellite instruments. We then examine early theoretical advances in retrieving soil moisture and snow properties, two applications that contributed to the future development of satellite microwave radiometry missions for the observation of surface variables. Particular attention is given to radiative transfer theory and its solutions, which model the effects of roughness, vegetation, and snow cover. These approaches form the basis of today’s retrieval algorithms and remain central to future missions. Subsequent sections highlight the use of passive microwave data for estimating a variety of surface variables, the role of passive microwave in data assimilation systems and forthcoming missions dedicated to land monitoring. The review concludes with key achievements, ongoing challenges, and open issues—such as soil moisture retrieval under dense vegetation or snow property retrieval in melting conditions. Addressing these limitations is critical to fully exploiting microwave radiometry in the context of climate research and mitigation strategies.

## 1. Introduction

Radiometers detect and measure the natural microwave emissions from the Earth’s surface. These emissions are expressed as brightness temperature (TB), which corresponds to the temperature of a blackbody that would emit the same radiation intensity as the observed surface. The relationship between brightness temperature and the actual physical temperature (T) of the surface depends on its emissivity (e)—a material property ranging from 0 (a perfect non-emitter) to 1 (a perfect emitter, or blackbody).

The microwave electric field often exhibits a preferred orientation, known as polarization, which is influenced by the geometric structure of the emitting or reflecting surface [1,2]. Due to the long wavelengths of microwaves and practical limitations on spacecraft antenna size [3], passive microwave radiometers generally have lower spatial resolution compared to instruments operating in the visible, infrared, or synthetic aperture radar (SAR) ranges. Typically, spaceborne passive microwave radiometers provide spatial resolutions between 10 and 100 km.

While the coarse resolution of the current passive microwave satellite missions might appear to be a limitation, it is not a major drawback for regional or global terrestrial biosphere models, which typically operate at spatial scales of several kilometers. In such models, capturing small-scale landscape variability (on the meter to kilometer scale) is often considered less critical than acquiring frequent observations. Moreover, in the applications of the past decades, the computational constraints limited the spatial resolution of models that assimilate passive microwave observations [4]. Nowadays, such computational issues are reduced and both the development of surface modeling and land surface microwave remote sensing are increasingly oriented toward achieving higher spatial resolution. At the same time, the future generation of passive microwave sensors (e.g., CIMR) will be able to guarantee a finer spatial resolution in low-frequency microwave observations compared to existing satellites. Furthermore, for many applications, the high temporal resolution of microwave instruments—often providing observations not affected by meteorological clouds every 2–3 days, with multiple daily overpasses in polar regions—is an important added value that compensates for the relatively low spatial resolution.

Another key advantage of passive microwave remote sensing is its ability to penetrate vegetation, soil, and snowpack, thanks to its longer wavelengths. This allows for subsurface observations, unlike visible and infrared sensors, which are limited to surface-level information [5]. Generally, the longer the microwave wavelength, the deeper the penetration. However, deeper penetration also complicates signal interpretation, as the received signal becomes a mixture of contributions from vegetation, soil, snow, and atmosphere [6,7,8].

To accurately extract information about surface conditions from these complex signals, the application of radiative transfer models is the widest and most consolidated approach. These computational tools simulate how electromagnetic waves interact with various surface components [9,10,11,12], accounting for processes such as scattering, reflection, and attenuation by elements like soil, vegetation, and snow.

By modeling the individual contributions of each surface component, radiative transfer models serve as powerful tools for disentangling the composite microwave signal [13]. This enables the retrieval of key environmental variables, such as soil moisture, vegetation properties, or snowpack characteristics. In essence, these models allow scientists to decode the complex information carried by microwave emissions and extract meaningful insights about land surface conditions.

Spaceborne passive microwave remote sensing offers unique advantages for monitoring land surfaces. Microwaves (wavelengths 1 mm to 1 m, frequencies 0.3–300 GHz) offer several benefits in observing land properties, particularly their ability to penetrate clouds and atmospheric attenuation (in the range of wavelength from ~8 mm to 1 m [14]), operating in almost all weather conditions, day or night [15]. Following World War II, the Institute of Electrical and Electronics Engineers (IEEE) established a standardized system for letter-designating radar-frequency bands of the microwave spectrum. These IEEE letter codes have since been widely adopted by engineers across various domains, including radar, satellite communications, remote sensing and terrestrial communication systems, providing a common, unambiguous language for specifying frequency ranges and facilitating global technical communication.

In Table 1 it is reported the IEEE band designations and their principal applications in remote sensing.

During the past decades, several satellite missions based on microwave radiometry have been developed by space agencies worldwide. In Table 2, a summary of the principal missions that provide official products on land surface parameters is reported.

## 2. From Theory and Experiments to First Applications

The discovery of the blackbody radiation theory gave rise to passive microwave applications between the 1860s and the 1940s. A number of investigations involving the first radiometers were conducted in order to determine the viability of this theoretical phase. These sensors may measure the Earth’s inherent thermal emission, as was previously noted, and provide information about the bulk dielectric and geometric features of the observed surface or volume.

Physicist Robert Dicke’s experiments in the 1940s marked the beginning of groundbreaking work in passive microwave radiometry. Although Dicke’s research during the war may have contributed to the specifics and applications, his creation of a microwave radiometer-receiver was a major advancement. The fundamental idea of passive microwave technology—the detection and measurement of naturally occurring electromagnetic radiation—was established by this device. There was a boom in exploration and development between 1950 and 1980. The first satellite experiment devoted to the observation of Earth covered by a passive microwave radiometer was made in 1968 by Basharinov et al. [16].

This was the foundation of satellite passive microwave remote sensing established by the pioneering experiment aboard the Soviet Kosmos-243 satellite, which employed multi-frequency microwave radiometry to simultaneously measure fundamental atmospheric and geophysical parameters globally. This initial methodology facilitated the world’s first retrieval of atmospheric total water vapor and cloud liquid water over oceans, successfully demonstrating the innovation of all-weather capability to detect and map precipitation and storm winds. Initial surface observations from Kosmos-243 also confirmed the strong influence of soil moisture and surface structure on emitted microwave radiation [17]. This early success paved the way for subsequent developments, most notably the Nimbus-5 Microwave Spectrometer (NEMS), which utilized microwave sounding channels to achieve the first successful retrieval of atmospheric vertical temperature profiles from space with an accuracy of ~2 K [18]. The NEMS mission further confirmed the technique’s all-weather robustness, providing fundamental geophysical outcomes on ice type, sea roughness, and snow cover. These foundational outcomes were soon applied specifically to terrestrial environments, with Schmugge et al. (1973 and 1974) providing key early work demonstrating the direct use of microwave radiometers for quantitative remote sensing of soil moisture [19] and snow and ice retrievals [20].

Retrospectively, Gorbunov and Kutuza (2018) [21] confirm that the physical principles and multi-channel methodology of Kosmos-243 represent the starting point for the entire field, serving as the innovation that underpins all modern microwave radiometers used in global weather and climate monitoring.

In the following years, researchers started employing this technology for Earth observation, concentrating on tasks like determining the amount of sea ice, soil moisture, and even atmospheric characteristics. The foundation for later satellite-borne microwave radiometers that would transform remote sensing capabilities was laid by these early investigations, which frequently used ground-based sensors or aerial platforms.

A milestone in soil moisture and snow monitoring has been achieved when considering the TB dependency not only on the moisture content of the soil or snow depth, but also on the contributions of the vegetation and atmosphere, the latter being negligible at certain wavelengths.

### 2.1. Soil Moisture First Applications

Early applied studies demonstrated the potential of passive microwave remote sensing to monitor surface soil moisture over land [19,22,23,24,25]. Variations in soil moisture across space and time are influenced by the heterogeneity of soil properties, topography, land cover, evapotranspiration, and precipitation. The interplay among these factors affects the electromagnetic signal detected by radiometers.

Soil moisture dynamics are governed by complex interactions within the soil–plant-atmosphere continuum [26,27], as part of the broader water cycle. An increasing number of satellite-based sensors are now being assimilated into models such as those from the European Centre for Medium-Range Weather Forecasts (ECMWF) and the Monitoring Atmospheric Composition and Climate (MACC) initiative [28].

The effectiveness of microwave remote sensing for retrieving soil moisture relies heavily on the distinct dielectric properties of water compared to those of soil particles. These properties are typically expressed in terms of the complex dielectric constant (ε), which characterizes a material’s response to an applied electromagnetic wave [29]. The dielectric constant is composed of a real part (ε′) and an imaginary part (ε″):

(1) $$\varepsilon =\varepsilon^{\prime }+{i\varepsilon }^{\prime \prime }$$

(2) $$\varepsilon_{r}=\frac{\varepsilon }{\varepsilon_{0}}$$

Here, $\varepsilon_{0}$ is the dielectric constant of free space. The real part ε′ governs wave propagation (e.g., velocity), while the imaginary part ε″ relates to energy loss or absorption as the wave travels through the medium [29,30]. In Equation (2) it is highlighted how the dielectric constant of a material ε can be expressed as a function of the deviation from the dielectric constant of the free space ($\varepsilon_{0}$) through the factor $\varepsilon_{r}$ which represents the relative dielectric constant of the material considered. At microwave frequencies, the contrast between the dielectric properties of soil and water is particularly pronounced.

For instance, dry natural soils exhibit ε′ values ranging from 2 to 5, largely independent of frequency, and ε″ values typically below 0.05 [5,31]. In contrast, the dielectric constant of water at 1 GHz and room temperature is approximately 80 (real part) and 4 (imaginary part) [32]. These substantial differences enable the use of passive microwave remote sensing to estimate soil moisture content, as even small additions of water can increase the dielectric constant of the soil–water mixture significantly—often exceeding a value of 20 [29]. However, the dielectric behavior of moist soil cannot be accurately represented by a simple weighted average of its constituents. Mixing models are required to describe the effective dielectric constant, which is influenced not only by water content and frequency, but also by other soil characteristics such as temperature, texture, and salinity [5,29]. For example, as soil temperature increases, ε′ tends to decrease due to reduced dipole alignment from increased thermal agitation.

Within the typical operational microwave frequency range (0.4–10 GHz), the dielectric constant is less directly affected by soil composition. Nonetheless, composition exerts an indirect influence via:

1. the amount of water bound to soil particles, which varies across soil types [31,32];
2. soil porosity, although its influence becomes negligible at volumetric moisture contents above ~5% [33].

In parallel, numerous studies have examined additional factors affecting microwave emissions, including vegetation cover [23,34,35], soil temperature [36,37,38], snow cover [39,40], as well as topography and surface roughness [24,41]. These factors can significantly influence the magnitude and characteristics of the emitted microwave signal from the land surface.

### 2.2. Snow

Due to the strong sensitivity of microwave emission signals to snow—particularly at 37 GHz—spaceborne microwave sensors have been widely used to estimate snow depth, snow water equivalent (SWE), and snow wetness [42,43,44,45,46,47,48].

In dry snow, upwelling microwave radiation is subject to scattering by snow crystals, with the degree of scattering influenced by snow depth and properties such as grain size and density [49]. Because brightness temperature is highly sensitive to changes in the dielectric constant, the microwave signal also responds strongly to variations in snow moisture content, making it possible to detect snowmelt onset and wet snow conditions [5,50,51].

To retrieve snow parameters, researchers have applied both physical microwave emission models (e.g., MEMLS—Microwave Emission Model of Layered Snowpacks) and empirical approaches that relate TB observations to ground-based measurements. Empirical approaches have demonstrated strong correlations between satellite-observed TB and in situ observations of SWE and snowmelt [43,45,46,47].

Microwave sensors are also effective for monitoring lake and river ice as well as sea ice. Passive microwave instruments, such as those on board the SSM/I and AMSR series, have been used to derive sea ice concentration, extent, type, and thickness, particularly in polar regions [52,53,54,55]. The capability of microwaves to penetrate cloud cover and operate independently of solar illumination makes them especially valuable for continuous monitoring in high-latitude regions during the polar night.

The polarization and frequency dependence of the microwave signal provide useful information about ice characteristics. For example, multi-frequency approaches combining 19 GHz and 37 GHz channels can distinguish between first-year and multi-year sea ice based on differences in emissivity and scattering behaviour [56]. Additionally, ice thickness retrievals over thin ice have been demonstrated using low-frequency microwave observations (e.g., 6.9 GHz), which are more sensitive to volume scattering and allow deeper penetration [57,58].

## 3. Radiative Transfer Theory

The theoretical foundation of radiometric missions is rooted in the development of radiative transfer (RT) theory, which provides a framework for describing microwave electromagnetic interactions between radiative energy and natural surfaces or volumetric scatterers, such as vegetation, soils, and snowpacks. These interactions are modeled using Microwave Emission Models (MEMs), which link the physical and dielectric properties of the medium to its microwave emission and scattering characteristics [5,59]. At the most fundamental level, Maxwell’s equations offer an exact representation of electromagnetic wave behavior for any geometry. However, directly solving these equations for natural, heterogeneous media is computationally prohibitive, especially when the number of scatterers is large and their spatial arrangement complex [59,60].

Following the heuristic derivation of the RT equation in the 1950s, researchers applied the theory to develop MEMs under statistically homogeneous assumptions for vegetation canopies. Such assumptions treat the structural components—branches, leaves, and trunks—as randomly distributed scatterers, enabling tractable solutions when detailed geometrical data are unavailable [61]. More recently, advances in computational electromagnetics have enabled the use of the Foldy–Lax Multiple Scattering Theory (MST-FL) to obtain exact solutions to Maxwell’s equations for multiple scatterers without invoking such homogeneity assumptions [62,63]. RT equations can be derived from MST-FL in two principal ways:

1. Heuristically, by applying the effective field approximation to Dyson’s equation and the ladder approximation to the Bethe–Salpeter equation [63,64,65], as originally suggested by Chandrasekhar in 1960 [66] and summarized later by Long and Ulaby [67].
2. Rigorously, by direct derivation from MST-FL without intermediate approximations.

In both derivation paths, only the dominant terms are retained [60,63,65,68]. However, the ladder approximation to the Bethe–Salpeter equation neglects cyclical (or closed-loop) scattering diagrams that contribute to coherent backscattering enhancement—an interference effect that increases the backscattered intensity in the exact backscatter direction [69,70]. As a result, standard RT equations cannot account for this enhancement unless additional terms or coherent scattering models are incorporated [65].

From a theoretical standpoint, MST-FL is rigorously derived by formulating the N-scatterer problem in Maxwell’s equations using integral equations, T-matrix methods, and the extinction theorem [63,71,72,73]. In this sense, solving the MST-FL equations is formally equivalent to solving Maxwell’s equations for multiple scattering systems. This exactness, however, comes at a high computational cost, motivating hybrid approaches that blend MST-FL rigor with the efficiency of RT-based approximations [73,74].

### 3.1. Principles and Applications

According to the Rayleigh–Jeans approximation, at microwave frequencies, the emitted power is approximately a linear function of the physical temperature of the target. Measuring this temperature allows retrieval of surface emissivity, which in turn provides insights into the intrinsic physical and dielectric properties of the observed medium. Because microwaves can penetrate below the immediate surface, they enable the extraction of both volumetric and near-surface geophysical parameters. In particular:

1. Volumetric information includes variables such as snow density and microstructure [75,76,77] and vegetation optical depth (VOD) [78,79,80,81].
2. Near-surface information includes soil moisture [6,82] and freeze–thaw state [83,84,85,86,87], owing to microwave sensitivity to the presence and phase of water.

The effect of vegetation on microwave emission varies with frequency, plant size, water content, and canopy density. At L-band, sparse vegetation such as croplands or grasslands generally behaves as a radiative attenuator. With increasing canopy dimension and density, vegetation also becomes a significant volume thermal emitter; in dense forests, canopy emission can dominate the measured brightness temperature. For L-band radiometers, dense forests may contribute more than ~85% of the total observed signal, leaving <~15% from attenuated soil emission. This partial retention of soil sensitivity is a distinct advantage of L-band over higher frequencies, where vegetation effects overwhelm the surface contribution.

Accurately modeling vegetation contributions is therefore critical for L-band missions such as ESA’s Soil Moisture and Ocean Salinity (SMOS) [27] and NASA’s Soil Moisture Active Passive (SMAP) [88], which retrieve soil moisture and VOD globally.

### 3.2. Zero-Order Solution of Radiative Transfer Equation for Vegetated Surfaces

In the simplest representation, the vegetation canopy is treated as a bulk attenuating layer, and scattering effects are parameterized using a single-scattering albedo. Under this framework, the emissivity of a vegetated surface is modeled as the sum of three components: (i) soil emission attenuated by the canopy, (ii) upward emission from vegetation, and (iii) downward emission from vegetation, reflected by the soil and further attenuated on its return path [5].

This approximation corresponds to the zero-order, non-scattering solution of the radiative transfer (RT) equation, in which scattering source terms are neglected [9]. The resulting tau–omega model describes vegetation effects using two parameters: the vegetation optical depth τ_p_, representing canopy attenuation, and the single-scattering albedo ω_p_, representing the fraction of intercepted radiation that is scattered rather than absorbed.

The τ−ω model formulation for the brightness temperature can be written as

(3) $${TB}^{p}=\left(1-\omega_{p}\right)\left(1-\gamma_{p}\right)\left(1+\gamma_{p}{\Gamma_{g}}^{p}\right)T_{C}+\left(1-{\Gamma_{g}}^{p}\right)\gamma_{p}T_{G}$$

The subscript p stands for polarization. T_G_ and T_C_ are the effective soil and vegetation canopy temperatures, Γ_g_^p^ is the soil reflectivity and γ_p_ is the vegetation attenuation factor. Consequently, in addition to soil reflectivity (Γ_g_^p^), three main effects can be distinguished in those of temperature (through both T_G_ and T_C_ terms), vegetation scattering (ω_p_) and attenuation (γ_p_).

**Figure 1:**
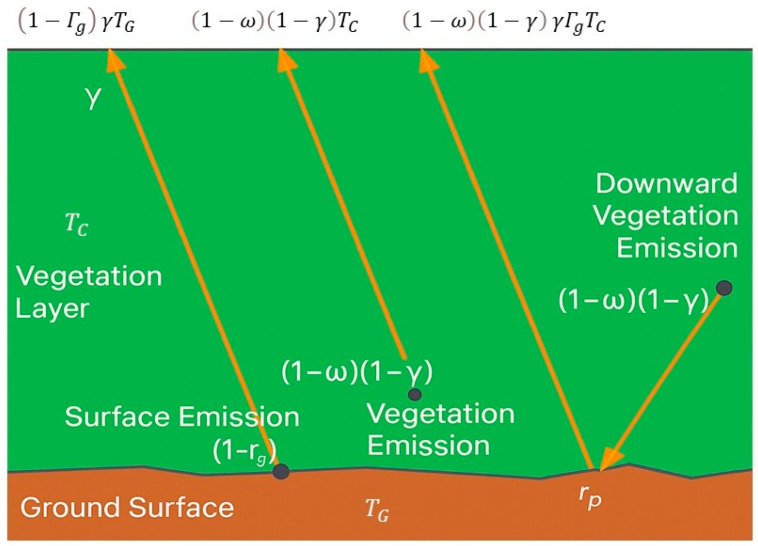
Schematic representation of the τ−ω model including the different contributions to the TB.

The vegetation attenuation factor can be computed from the nadir optical depth τ_p_ as:

(4) $$\gamma_{p}=exp(\frac{-\tau_{p}}{cos\theta })$$

and it is related to the vegetation properties and the frequency. The single scattering albedo describes the scattering of the emitted radiation by the vegetation, and is a function of plant geometry. A schematic representation of the τ−ω model is reported in Figure 1.

Physically, τ_p_ is related to vegetation biomass, water content, and structure [89,90,91,92], while ωp reflects leaf and branch geometry, orientation, and dielectric contrast. These parameters are central to operational retrieval algorithms, linking measured brightness temperatures to biophysical variables across diverse land cover types.

While the tau–omega model provides a computationally efficient first-order approximation, more advanced formulations extend its capability to account for multiple scattering, polarization dependence, and coherent effects. For example, multi-order scattering can be incorporated through iterative solutions of the RT equation or by embedding the tau–omega structure within the Vector Radiative Transfer Equation (VRTE) to handle cross-polarized terms [59,93]. In dense vegetation or forested regions, where scattering is non-negligible, these advanced models integrate canopy structural information—such as leaf angle distribution, clumping index, and vertical layering—either through analytical phase functions or through numerical methods like the Discrete Ordinate Method (DOM) and Monte Carlo simulations [74,94]. Such refinements bridge the gap between simple parameterized models used in global retrieval algorithms and full-wave electromagnetic solutions, offering improved accuracy in environments where the basic tau–omega assumptions break down. These more advanced approaches are presented in the next subsection.

### 3.3. Scattering Solution of Radiative Transfer Equation for Vegetated Surfaces

Building on the zero-order RT formulations discussed earlier, the discrete-scatterer approach can be viewed as a more physically correct evolution of the tau–omega model. While the tau–omega parameterization treats the vegetation layer as a homogeneous attenuating slab characterized by bulk optical depth and single-scattering albedo, the discrete model resolves these bulk parameters into contributions from physically distinct canopy elements. This connection ensures theoretical consistency with classical RT while enabling the direct use of measurable biophysical variables—such as leaf water content, stem diameter, or needle orientation—in forward simulations. Consequently, discrete-scatterer models provide a pathway to integrate fine-scale canopy structure into passive microwave emission modeling, while retaining compatibility with operational inversion schemes derived from simplified RT theory.

A more physically rigorous approach to calculating microwave emissions from vegetated surfaces is based on Radiative Transfer (RT) theory, which accounts for both single and multiple scattering phenomena. Within this framework, the vegetation layer is modeled as an ensemble of discrete components—leaves, branches, stems—whose statistical distributions in position, size, and orientation are prescribed. Because RT assumes independent scattering, it neglects phase coherence and therefore treats only incoherent radiation. Despite this limitation, the conceptual simplicity and computational efficiency of RT have made it a cornerstone in passive microwave remote sensing of vegetation. A substantial body of literature addresses the interpretation of radiometric measurements in terms of geophysical parameters by solving the inverse problem of the RT equations.

This “discrete scatterers” representation assigns each vegetation element a specific geometry and dielectric constant distinct from the surrounding air [63,95]. Idealized canonical shapes are typically adopted: thin dielectric disks for leaves, finite-length dielectric cylinders for stems, branches, and needles. Analytical and approximate scattering models for these shapes have been developed extensively [96,97,98,99]. The single-scattering characteristics of these elements—averaged over their statistical distributions—govern the canopy’s effective attenuation and scattering properties. This direct link between scattering parameters and vegetation biophysics enables the retrieval of properties such as water content, size distribution, and orientation from radiometric data.

In the general case of a vegetated soil surface, retrievals rely on the selection of an appropriate forward model and its inversion. An ideal forward model represents all major processes contributing to the observed emission, encompassing the dielectric and geometric characteristics of both soil and vegetation. However, for operational purposes, simpler forward models are often preferred to ensure computational tractability, even at the cost of neglecting certain structural or dielectric details (e.g., exact element shapes, size variability, or anisotropic permittivity).

Discrete models remain widely used for vegetation because they (i) capture the essential electromagnetic properties of individual elements, (ii) can be combined with advanced soil scattering theories, and (iii) require inputs that can be reasonably measured in the field. Their development generally follows three steps:

1. Canopy decomposition into individual elements with selected canonical shapes.
2. Electromagnetic characterization of each element, including its complex permittivity, extinction cross section, and scattering cross section.
3. Integration of contributions from all elements to simulate the overall microwave emission and scattering from the canopy–soil system.

In these models, the soil is typically treated as a homogeneous half-space with a rough surface producing diffuse scattering—a standard assumption across most formulations. Stems or trunks are represented as near-vertical dielectric cylinders, with a single cylinder assigned per stem; twigs and petioles are also commonly modeled as small cylinders [100]. Crop or deciduous tree leaves are approximated as dielectric disks, with circular disks suitable for small leaves (e.g., alfalfa, soybean) [101,102], while long leaves (e.g., wheat, corn) may require curved-sheet models to capture bending and surface irregularities [103]. A simpler alternative, often used at L-band and below, is to represent these long leaves as multiple small disks [100,104]. Needle-like leaves of conifers are modeled as thin prolate ellipsoids; seed heads and pods may be represented as dielectric cylinders [105,106], though in some models these are omitted entirely.

The statistical distributions of element dimensions and orientations depend on vegetation type and phenological stage, and may be derived from growth models [100] or allometric relationships [107,108]. Comprehensive reviews of electromagnetic approximations for vegetation are provided by Karam et al. [99] and of land-surface radiometry models by Wigneron et al. [13,109].

Most discrete vegetation models compute element permittivity using the semi-empirical formulation of Ulaby & El-Rayes [110], which relates complex dielectric constant to volumetric moisture content, free- and bound-water fractions, and free-water ionic conductivity. These parameters were originally determined through regression fits to laboratory measurements. Mätzler [10] later extended the model by combining these data with new permittivity measurements over a wider range of vegetation types and leaf structures. While the permittivity estimation and the calculation of scattering/extinction cross sections are similar across discrete modeling approaches, the methods used to integrate the contributions from different element classes vary significantly.

Some studies have adopted coherent combination methods for element contributions [111], which require detailed knowledge of scatterer positions and orientations, and generally remain limited to first-order scattering. Although such methods can, in principle, capture interference effects absent in conventional RT, their practical application is restricted by the difficulty of obtaining the necessary canopy-structure inputs over large areas.

A straightforward yet effective method for modeling vegetation scattering is based on a first-order formulation of Radiative Transfer (RT) theory [112,113,114]. In this approach, an incident electromagnetic wave interacting with vegetation elements undergoes three primary processes: backscattering, specular scattering (downward scattering toward the ground in the mirror-reflection direction relative to the incidence angle), and attenuation. The overall scattering and attenuation properties of the vegetation layer are obtained by incoherently summing the individual contributions of all canopy elements—averaged over their size and orientation distributions. For the underlying soil, only backscattering and specular scattering are considered. A well-known implementation of this approach is the Michigan Microwave Canopy Scattering Model (MIMICS) [61], originally developed for forest environments and validated over the 0.5–10 GHz frequency range at incidence angles greater than 10° [115,116].

The first-order approach offers the advantage of relative simplicity, as scattering is modeled in only two directions (backward and specular). However, its main limitation lies in the neglect of multiple scattering effects.

**Figure 2:**
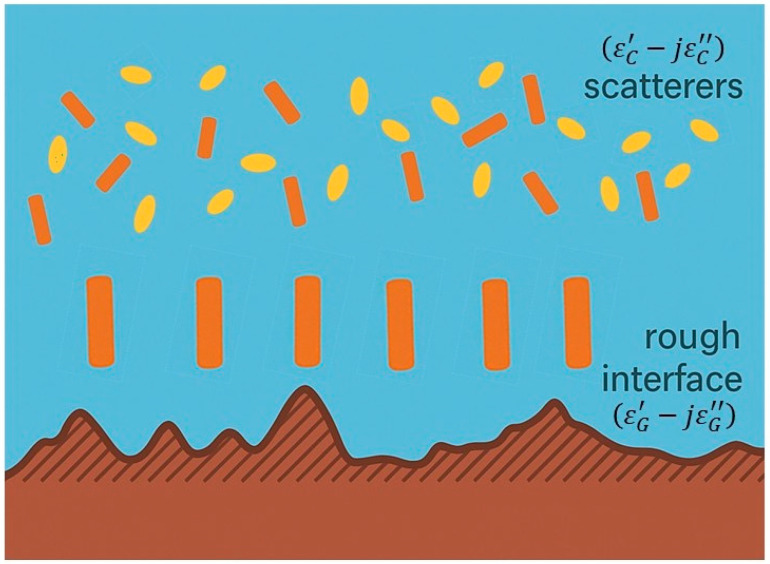
Model of a vegetation layer over soil.

To account for multiple scattering of any order, the Matrix Doubling algorithm provides a robust solution. Initially developed by Twomey et al. [117] to model scattering in atmospheric media under the Rayleigh approximation, the method was later adapted to ensembles of dielectric discs—representing leaves—above soil surfaces [118]. This framework forms the basis of the electromagnetic model developed at the University of Tor Vergata, which can represent complex canopies composed of leaves, stems, and petioles over soil [102] as well as forested environments [119]. In the latter, the tree’s elements are modelled by discs (for leaves) and cylinders (for stems, branches and trunks) as shown in Figure 2, where also the dielectric dependences on the dielectric properties of the canopy and the underlying rough soil surface are highlighted.

In this formulation, the vegetation layer is divided into thin sub-layers where interactions between scatterers within the same layer are negligible. For each sub-layer, scattering and transmission matrices are computed based on the bistatic scattering cross sections of the contained vegetation elements, averaged over their dimensions and orientations. The Matrix Doubling method is then applied iteratively to combine the matrices of successive sub-layers, yielding the overall scattering and transmission properties of the entire vegetation layer. A similar matrix representation is derived for the soil, proportional to its bistatic scattering coefficient, and subsequently combined with the vegetation response using the same doubling procedure.

This approach inherently captures multiple scattering interactions between vegetation components and between vegetation and soil. For tree trunks, only the double-bounce effect in the incidence plane and attenuation are considered, since their vertical orientation and large dimensions produce strongly peaked scattering in the downward specular direction. For crop canopies with near-vertical stems, the full bistatic scattering pattern is simulated. In these cases, a second lower layer is introduced so that, in addition to the double-bounce effect, non-specular interactions with the rough soil surface are also incorporated into the model.

Energy conservation is used to simulate passive measurements [120], so that the emissivity e(θ_i_,p_i_) at a specific angle θ_i_ and polarization pi complement reflectivity as:

(5) $$e_{p}(\theta_{i})=1-\frac{1}{4\pi \cos\theta_{i}}\int (\sigma_{pp}^{0}(\theta_{i},\theta_{s},\phi_{s})+\sigma_{pq}^{0}(\theta_{i},\theta_{s},\phi_{s}))d\Omega_{s}$$

where reflectivity is the integral of the bistatic scattering coefficient (in both co- (pp) and cross-polarization (p_q_)) over all scattering directions [5,104].

### 3.4. Relevant Adaptations for Vegetated Surfaces

The scarcity of experimental datasets, combined with the inherent complexity of single and multiple scattering processes within forest canopies, has encouraged the adoption of theoretical modeling as a tool for parameterizing simplified zero-order models. In Europe, this approach gained momentum through the development of a forward model to support the retrieval of observations from the SMOS mission [6]. Among the available candidates, the Tor Vergata University theoretical emission model—based on a discrete representation of vegetation canopies and explicitly accounting for multiple scattering effects—was selected [93].

The methodology for fitting the parameters of a zero-order radiative transfer model using this theoretical framework was outlined in Ferrazzoli et al. [120]. The coniferous Landes Forest served as the reference site. Brightness temperatures were simulated for various stages of forest growth—expressed in terms of vegetation water content (kg m^−2^)—across defined ranges of soil moisture and observation angles. The results indicated simulated L-VOD values between 0 and 0.8, consistent with later studies. The model also predicted relatively high albedo values, ranging from 0.10 to 0.15, depending on branch orientation. This preliminary analysis faced limitations due to the scarcity of validation measurements and the lack of detailed forest structural data, such as branch size and orientation distributions, which significantly influence emissivity and are required as inputs for the theoretical model. These constraints were subsequently addressed in follow-up studies—most notably Saleh et al. [108]—and the model was later successfully validated against airborne radiometric measurements [121,122]. In parallel, simulation-based calibration of the zero-order model parameters was performed for both coniferous and deciduous forests, with the resulting parameter sets validated against ground-based radiometric observations [123].

In the U.S., the applicability of the zero-order radiative transfer (RT) model for representing forest microwave emission was evaluated by Kurum et al. [124]. In that study, the optical depth (τ) and single scattering albedo (ω) were directly retrieved by fitting multi-angular L-band radiometric measurements collected over a coniferous forest site in Maryland. These retrievals were then compared with (i) theoretical simulations from a first-order RT model that included multiple scattering effects, and (ii) active transmissivity measurements obtained using an L-band radar and a corner reflector. The comparison showed strong agreement and confirmed that the retrieved albedo should be interpreted as an effective parameter, which was found to be significantly lower than the intrinsic single-scattering albedo of individual branches.

### 3.5. RT Solutions for Snow Covered and Ice Surfaces

The successful application of radiative transfer (RT) models to forest canopies—ranging from simple τ–ω parameterizations to advanced multiple-scattering simulations—has highlighted the flexibility of the RT framework in representing complex natural media. Although the scattering elements and dielectric properties differ greatly between vegetation and other surfaces, the underlying challenge remains the same: accurately capturing how a heterogeneous medium modifies microwave radiation through absorption, emission, and scattering. This conceptual continuity has facilitated the extension of RT theory to cryosphere studies, particularly in modeling snow-covered terrains. In snowpacks, the vegetation “scatterers” are replaced by snow grains, and the canopy attenuation and scattering processes find their analogue in the volume scattering and absorption caused by the snow microstructure. By adapting the same principles—while accounting for the unique grain-size-dependent Mie scattering regime and the influence of liquid water—researchers have developed RT-based snow models capable of describing the combined emission from both the snow volume and the underlying ground.

Research into snow monitoring using passive microwave observations advanced during the same period as studies focusing on soil moisture and vegetation effects on radiometric signals. The microwave emission from a snowpack is primarily composed of two components: (i) volume emission from the snow itself and (ii) emission from the ground beneath the snowpack. Because dry snow has inherently low emissivity, a significant portion of the observed microwave radiation originates from the underlying ground. The scattering behavior of the snowpack—which is sensitive to grain size distribution, snow depth, particle shape, and moisture content—provides the physical basis for snow detection using passive microwave radiometry. The dielectric properties of snow grains and the presence of liquid water strongly influence propagation, scattering, and attenuation within the snowpack [125,126].

In RT modeling, snow grains are typically represented as randomly distributed spherical particles that induce non-coherent scattering. Because the size of snow grains is often comparable to the wavelength of the microwaves under consideration, Mie scattering is an important process [127]. Microwave radiation emitted from the ground beneath the snowpack undergoes both volume scattering and absorption during propagation through the snow, with the magnitude of these effects depending on snowpack properties. In dry snow, the dielectric contrast between air and snow grains causes significant dispersion [128], leading to internal volume scattering at wavelengths similar to the grain size and a corresponding reduction in passive microwave emission, while the presence of liquid water introduces strong absorption and changes in the effective dielectric constant [126], resulting in a rapid increase in emissivity and a reduction in penetration depth.

At longer wavelengths (e.g., ≥1.4 cm, or ≤18 GHz), the interaction between snow particles and microwave energy is reduced, allowing for higher emissivity and greater transmission regardless of snow depth. In contrast, thicker and denser snowpacks contain a higher concentration of scattering particles, increasing absorption and reducing emission, particularly at shorter wavelengths [129].

The penetration and scattering characteristics of snow are highly sensitive to microstructural parameters such as grain size, shape, and density. For instance, larger grain sizes and higher densities increase scattering at high frequencies (≥18 GHz), thereby reducing surface-transmitted emission from the ground [130,131]. At lower frequencies, including L-band (1.4 GHz), volume scattering is generally weaker, allowing deeper penetration and a more significant ground contribution. However, in thick or dense snowpacks, even low-frequency microwaves can experience appreciable attenuation [132].

The RT approach for snow has been expanded to incorporate multiple layers of differing grain size, density, and liquid water content, as in the MEMLS (Microwave Emission Model of Layered Snowpacks) [131], which solves the RT equation for layered media with internal scattering and reflection. This model has been successfully applied in both dry and wet snow conditions and has been extended to include surface roughness effects and ice crust layers. Other works have adapted the Dense Media Radiative Transfer (DMRT) theory [132,133] to snow, explicitly accounting for particle correlation functions and non-spherical grain shapes, which are important in aged snow or firn.

For glacial ice, the same principles apply, but with larger and more complex scattering structures, including air bubbles, brine pockets, and crystal boundaries [134,135]. In this case, the RT modeling must account for strong anisotropy in dielectric properties due to preferential crystal orientation and the possibility of coherent effects over long path lengths. These adaptations have proven essential for applications such as estimating ice thickness, detecting melt onset, and retrieving snow accumulation rates over polar ice sheets, as well as for quantifying liquid water in melting conditions [136].

### 3.6. Summary of RT Approaches for Vegetated and Snow/Ice Covered Surfaces

Overall, the adaptation of RT theory from vegetation to snow and ice demonstrates the robustness of the framework, provided that the scattering elements and their statistical distributions are correctly parameterized. In both domains, the main limitation remains the availability of accurate microphysical input parameters—such as grain size distribution, density profiles, and liquid water fraction—which are critical for constraining the models and for successful retrievals from passive microwave measurements. In Table 3 the key features of the RT Modeling Approaches for vegetation, snow, and ice surfaces are reported.

## 4. Passive Microwave Remote Sensing for Retrieval of Surface Variables

Earth’s surface is a constantly evolving mosaic of landscapes, each possessing distinct physical and biogeochemical properties that shape and regulate global climate and environmental processes. Accurate monitoring of key surface variables—such as soil moisture, snow cover, vegetation biomass, and surface temperature—is vital for a broad spectrum of scientific and societal applications, including weather forecasting, climate change assessment, drought monitoring, and sustainable water resource management. Passive microwave remote sensing has emerged as a particularly powerful tool in this regard, owing to its ability to penetrate cloud cover, operate under all weather conditions, and provide consistent, large-scale observations both day and night. These capabilities enable detailed investigation of processes linking terrestrial water, energy, and carbon cycles, facilitate the estimation of global water and energy fluxes at the land surface, and support quantification of net carbon fluxes in boreal and other critical ecosystems. Furthermore, the integration of passive microwave data into numerical models enhances weather and climate forecasting skill, strengthens flood prediction systems, and improves drought monitoring capabilities, ultimately contributing to more effective environmental management and disaster preparedness on a global scale.

In addition to these applications, microwave radiometry plays a fundamental role in the monitoring of Essential Climate Variables (ECVs) as defined by the Global Climate Observing System (GCOS). Long-term passive microwave observations have been instrumental in deriving global records of soil moisture [137], snow cover and SWE [138], vegetation optical depth as a proxy for biomass [90,92,139,140], and land surface freeze–thaw dynamics [87]. These variables are critical for understanding land–atmosphere interactions, constraining carbon and water budgets, and evaluating climate feedback mechanisms. Recent advances in multi-sensor harmonization [141,142] have extended the temporal consistency of ECV datasets, allowing researchers to assess long-term variability and detect emerging trends linked to climate change. Importantly, passive microwave observations are increasingly being assimilated into land surface and climate models, improving the representation of soil moisture, snow dynamics, and vegetation processes in Earth system simulations [143,144,145]. Such assimilation efforts not only enhance short-term prediction skill but also contribute to reducing structural uncertainties in long-term climate projections, strengthening the scientific basis for climate change assessments.

As a result, microwave radiometry underpins many of the global climate data records that serve as benchmarks for international climate assessments and policy-making, reinforcing its central role in Earth system monitoring.

### 4.1. Soil Moisture

The sensitivity of microwave signals to surface soil moisture content—defined as the amount of water bound to soil particles in the upper few centimetres of the land surface—has been well established in previous research.

Yet key challenges persist, notably the parameterization of organic/peat soil dielectric behaviour [146,147], the treatment of vegetation attenuation and surface-roughness-induced scattering [148,149]. Recent work has revisited organic soil dielectric models and highlighted the need to explicitly account for soil organic matter to avoid bias in L-band retrievals [150,151]. Advances in canopy radiative transfer have also targeted the τ–ω framework, proposing refinements and new diagnostics to balance scattering and optical depth in vegetated landscapes, including forests [152,153,154]. Among microwave frequencies, L-band remains the most sensitive to soil moisture because longer wavelengths probe deeper into the soil and are less affected by moderate vegetation cover [155]. In practice, passive microwave retrievals constrain moisture in roughly the top ~5 cm of soil, with effective penetration depth modulated by frequency, moisture state, and vegetation characteristics.

Driven by agricultural and hydrometeorological applications, decades of algorithmic development at C/X bands using AMSR-E/AMSR-2 laid the groundwork for today’s L-band missions [156,157,158,159]. Passive L-band satellites—NASA’s SMAP and ESA’s SMOS—were conceived specifically to improve soil moisture estimation [6,89]. The retrieval problem is rooted in radiative transfer and requires land surface temperature, vegetation optical depth/scattering terms, roughness, and soil dielectric properties as inputs. While global L-band products historically featured footprints on the order of ~36–40 km, the community has pushed toward finer scales via enhanced processing and downscaling. Notably, SMAP’s enhanced products and multi-sensor fusion/downscaling approaches now target ~9–10 km [160,161,162]. Parallel efforts quantify retrieval uncertainty and seasonal error structures to improve multi-sensor merging and assimilation [163,164].

Compared with C/X-band, L-band brightness temperatures better sense deeper soil layers and retain skill under moderate vegetation, yielding the most accurate satellite soil moisture retrievals to date. Contemporary global products are primarily derived from SMAP and SMOS; evaluations continue to report very good performance from L2/L3 algorithms, demonstrating high accuracy in soil moisture estimates including over forests [165], as validated against in situ measurements and rainfall data, including over forested areas [166]. Nevertheless, retrievals under dense forest canopies remain challenging [90,167] with a sensible decrease in the average statistics (e.g., ubRMSD and R), achieved by comparing satellite retrievals of L-band SM with in situ measurements from ground networks over North America, in particular for forest sites at high latitudes [149,168]. Additional challenges are related to the observations over wetlands and mountainous terrains. In wetlands, the presence of standing water and variable inundation levels strongly alter the dielectric properties of the surface [169], masking the soil signal and leading to large retrieval uncertainties. The complex interactions between open water, saturated soils, and vegetation canopies further complicate the separation of soil moisture contributions from surface water effects, as reported in analyses of SMOS data [6]. Similarly, mountainous regions degrade retrieval accuracy due to steep topography, which introduces variable incidence angles, the rotation of the radiation polarization plane, shadowing, and mixed-pixel effects within the radiometer footprint [170,171,172]. Studies using SMAP observations have highlighted how snow cover, dense forests, and heterogeneous terrain exacerbate the difficulty of modeling surface emissivity, thereby limiting retrieval reliability [88,173]. Consequently, both wetlands and mountainous terrains remain critical sources of uncertainty in passive microwave soil moisture products, motivating the development of specialized retrieval algorithms such as the example of Bai et al. [174] and the integration of ancillary datasets to enhance retrieval performance in these complex environments.

Ongoing reprocessing and algorithm updates (e.g., SMAP data set updates at NSIDC or the next reprocessing of ESA SMOS L2 products) and new fused L-band products are further improving temporal continuity and consistency for modeling and data assimilation applications [13,109,175].

Passive microwave radiometers typically retrieve surface soil moisture with ubRMSE ≈ 0.03–0.11 m^3^/m^3^ and correlations R ≈ 0.4–0.85 depending on sensor, region, vegetation, and algorithm; L-band missions (SMOS, SMAP) achieve the best overall performance.

Factors impacting the accuracy of passive microwave soil moisture retrievals are multifactorial, stemming from environmental conditions and retrieval algorithm complexities. These factors directly explain the observed ranges in validation metrics, such as unbiased Root Mean Square Error (ubRMSE) and correlation (R). Vegetation cover significantly degrades radiometer sensitivity to surface soil moisture, often leading to underestimation or larger ubRMSE in vegetated regions, a trend notably observed with SMOS as vegetation increases [165,176]. Similarly, surface roughness alters surface emissivity and can introduce biases, especially when roughness parameters are uncertain; hence, accounting for or jointly estimating roughness can mitigate RMSE in passive retrievals [176,177]. The sensor frequency and penetration depth are also critical; L-band (1.4 GHz) offers superior penetration and reduced sensitivity to vegetation, typically achieving the best global ubRMSE (around 0.03–0.05 m^3^/m^3^ in many validations), whereas higher-frequency C/X-band radiometers exhibit larger errors over vegetated areas [165,176]. Furthermore, environmental phenomena like frozen ground, snow cover, ice, and radio-frequency interference (RFI) necessitate screening or flagging, as they cause retrieval failures or diminished skill, consequently affecting data coverage and effective accuracy [177,178]. The inherent spatial heterogeneity and footprint mismatch between coarse radiometer footprints (tens of km) and localized in situ point networks introduce a representativeness error, artificially inflating the apparent RMSE unless sophisticated downscaling or accounting methods are employed [170,177]. Finally, the accuracy depends on ancillary inputs and parameterization, such as Vegetation Optical Depth (VOD) and surface temperature. Variations in VOD parameterization, for instance, are known to generate systematic wet/dry biases across different sensor products like AMSR2, SMOS, and SMAP [2,179]. Collectively, these factors elucidate the variability in ubRMSE and R documented across different regions, seasons, and sensors in the soil moisture validation literature [176,177].

The inherent limitations and scale of passive microwave soil moisture retrievals dictate mission-level accuracy, with constraints stemming primarily from spatial resolution, representativeness, and product screening. The coarse native resolution of sensors like SMOS and SMAP, with footprints typically ranging from 25 to 40 km, necessitates significant spatial averaging. While downscaling techniques, often to 1 km, aim to enhance local usability, they can sometimes increase apparent error or rely heavily on auxiliary assumptions; for instance, global downscaling of SMOS has reported an average ubRMSE of ≈0.114 m^3^/m^3^ over multiple sites [177]. Conversely, downscaling or fusion approaches may improve skill relative to the coarse product, depending on the specific method and region, though they fundamentally alter the error characteristics. Another significant constraint is representativeness error. While comparisons at well-established core validation sites using appropriately representative ground networks often yield consistent ubRMSE values, sparse in situ networks can introduce bias into evaluation statistics, especially over regions with high footprint heterogeneity [165]. Furthermore, systematic biases by land cover and season are commonly reported in intercomparison studies. These biases are region- and season-dependent, encompassing large winter biases or errors in monsoon transitional zones, which implies that local deviations from globally typical RMSE ranges should be anticipated [4,176]. Practically, the best L-band radiometer performance ubRMSE ≈ 0.03–0.05 m^3^/m^3^) is generally expected in low-to-moderate vegetation or grassland areas, while performance significantly degrades ubRMSE ≈ 0.08–0.11 m^3^/m^3^ or larger) over dense vegetation, snowy/frozen areas, or strongly heterogeneous landscapes [176,177,180]. Table 4 compares representative validation results reported for SMAP, SMOS, and AMSR- E/AMSR-2 from multi-site and campaign evaluations.

### 4.2. Surface Temperature

Microwave brightness temperature (TB) is theoretically linked to the effective surface temperature T_eff_, enabling the retrieval of Land Surface Temperature (LST) from radiometric observations [181]. In practice, however, LST estimation from microwave data is more challenging and generally less precise than retrievals from thermal infrared (TIR) sensors. While state-of-the-art TIR retrievals can achieve accuracies of 0.2–2 K under favorable conditions [182,183], microwave-based approaches typically yield accuracies in the range of 1–5 K, depending on sensor frequency, surface type, and atmospheric conditions. This is partly due to the higher sensitivity of microwave emissivity to environmental variables—particularly surface liquid water content, vegetation water content, and soil roughness—compared to TIR emissivity.

Despite this lower nominal accuracy, microwave LST retrieval offers distinct advantages. Microwaves penetrate cloud cover and are largely unaffected by atmospheric aerosols, making them invaluable when TIR observations are obscured. Moreover, the longer wavelengths of passive microwave sensors allow limited penetration into the subsurface (a few centimeters), enabling retrieval of ground temperature below the immediate surface. This property has been exploited for monitoring subsurface thermal regimes beneath snow and ice [184,185], a capability that TIR sensors inherently lack. Recent studies have demonstrated the feasibility of retrieving snow–soil interface temperatures using L-band radiometry from SMOS and SMAP [186,187], improving the representation of snow thermal insulation in climate models.

Although no operational, globally distributed microwave LST products are yet available, algorithmic progress is accelerating. Current retrieval methods, drawing on data from AMSR2, SMAP, SMOS, and GPM Microwave Imager (GMI), typically achieve accuracies of 2–5 K [183,188]. Machine learning approaches have shown promise in narrowing this gap: neural-network-based retrievals using AMSR2 TB and ancillary datasets have reached 1–3 K accuracy in validation studies [189,190]. More recently, deep learning frameworks integrating multi-frequency microwave data and reanalysis fields have achieved sub-2 K accuracy in certain environments [191,192]. These methods also offer the potential for diurnal LST monitoring by leveraging the regular overpass schedules of multiple microwave satellites.

Emerging work is exploring synergistic retrievals that fuse TIR and microwave datasets, exploiting the all-weather capability of microwaves and the high precision of TIR to produce gap-free, high-accuracy global LST products [193,194]. Such hybrid products are expected to be especially beneficial for agricultural drought monitoring, polar region energy balance studies, and climate model data assimilation—applications where continuous LST coverage is critical.

In the context of ice sheets, microwave radiometry provides a unique capability for estimating surface temperature where thermal infrared sensors are often limited by persistent cloud cover and polar night conditions. The effective surface temperature retrieved from microwave brightness temperatures has been successfully applied to monitor ice sheet thermal dynamics, with studies demonstrating that L-band and higher-frequency radiometers capture both surface and near-surface thermal states [195,196,197]. Unlike TIR sensors, which measure only the skin temperature, the penetration depth of microwave signals (ranging from millimeters at high frequencies to several centimeters at L-band) allows for the characterization of subsurface snow and firn layers, offering insights into thermal storage processes and energy exchange within the ice sheet. This property is particularly valuable for assessing surface melt onset and refreeze events, which are key indicators of ice sheet mass balance and surface energy budget variability [198,199]. However, retrieval accuracy remains challenged by the influence of snow microstructure, liquid water content, and layering, which alter emissivity and can bias effective temperature estimates. Recent algorithmic advances using SMOS, SMAP, and AMSR2 observations, combined with radiative transfer modeling and machine learning techniques, are helping to refine retrievals and better represent ice sheet thermodynamics in climate models [200,201].

Passive-microwave surface temperature retrievals typically show errors from ~1–2 K for advanced ML/physical approaches under favourable conditions to ~5 K (or larger biases) for some physically based global products; accuracy strongly depends on surface type, atmosphere, and algorithm choice.

Validation results for passive microwave temperature retrievals exhibit strong dependence on the underlying surface type. Retrieval accuracy generally varies with vegetation density. Over forests, single-channel 37 GHz retrievals often demonstrate good precision, typically less than 2.5 K [202]. In moderately vegetated areas, more sophisticated methods, such as neural networks and coupled approaches utilizing AMSR2 data, have achieved mean errors in the range of ≈1.4–1.8 K [203,204]. Conversely, over low vegetation and sparse cover, retrieval precision tends to degrade compared to dense vegetation. For instance, single-channel methods over low vegetation have been reported to yield errors of ≤3.5 K [202]. Furthermore, physically based global algorithms show a tendency to overestimate Land Surface Temperature (LST) most strongly over barren or sparsely vegetated surfaces, resulting in larger biases and RMSEs in these areas [205].

Specific surface conditions present unique challenges. Over arid, semi-arid, and bare soils, the difficulty in discriminating between soil moisture and temperature significantly hinders performance. Iterative inversions using 6–18 GHz channels, for example, show worse performance for bare soils, which are often cited as an exception to the general retrieval goal of ≈2 °C accuracy [206,207]. In the case of snow and sea ice, the large emissivity variability of these surfaces under cold conditions is a primary source of retrieval uncertainty. Global microwave LST methods consistently encounter difficulties and larger errors when applied to these snow and ice conditions [208,209]. Finally, for open water or sea surface temperature (SST) retrievals, high accuracy can be achieved by combining multiple channels with machine learning (ML) techniques. Recent studies show that ML approaches for SST achieve errors that are competitive with established methods, reflecting high performance potential in this domain [203,210].

The spread in the literature’s reported accuracies for passive microwave temperature retrievals arises from a complex combination of physical and methodological factors. The single most dominant source of error is often surface emissivity variability and type. Differences in emissivity (e.g., between first-year and multi-year ice, or bare soil and vegetation) directly translate to errors; specifically, percentage errors in emissivity map to comparable percentage errors in the retrieved temperature in microwave sea-ice/ice-pack studies [209].

Several environmental conditions significantly modulate the microwave signal. Soil moisture coupling presents a major challenge because microwave emissivity is strongly modulated by surface moisture. The difficulty in separating the thermal signal from the moisture effect directly increases errors, particularly over bare soils and sparsely vegetated areas [204,206]. Atmospheric effects and Precipitable Water Vapor (PWV) also play a role; including PWV estimation and atmospheric correction schemes can significantly improve surface/air temperature retrievals. For example, AMSR2 schemes that retrieve PWV first have reported high correlation (R^2^ > 0.80) for surface air temperature retrievals [211]. Although ‘s ability to penetrate clouds provides an all-weather advantage where TIR fails, the presence of clouds still affects radiative signals, meaning accuracy depends heavily on appropriate channel selection and robust atmospheric correction [208,212]. Furthermore, snow and ice conditions introduce large variability in microwave emissivity under cold conditions, which causes systematically larger RMSEs and biases in global products over polar and high-latitude regions [208,209].

The physical properties of the sensor and the methodology of the algorithm introduce additional variability. Surface heterogeneity and footprint pose a validation challenge; the coarse footprint averages heterogeneous surfaces, meaning validation against high-resolution, point-based in situ sensors can show artificially inflated errors due to a representativeness mismatch [206]. The choice of sensor frequency, polarization, and incidence angle critically affects the retrieval. For land LST, 37 GHz is often favored; its vertical polarization balances lower surface sensitivity with higher atmospheric transmissivity, which leads to better theoretical bias and precision for vegetated areas [202]. Regarding the retrieval process itself, algorithm training and climatology are key. Machine learning or neural network approaches achieve low errors only when trained on representative TIR or in situ labels but suffer significantly if the training datasets do not span the target conditions. Conversely, physical-iterative methods rely on the accuracy of the forward model and the characteristics of the calibration regions used [204,212,213].

Finally, accuracy is highly contingent upon the validation design, specifically the reference target used (e.g., MODIS LST, ground stations, radiosondes, or reanalysis). Direct comparisons to MODIS, for instance, can show different bias patterns than those from point-station comparisons. For example, some physically based LSTs have been documented to overestimate versus MODIS by an approximate ≈3 K bias and ~5.4 K RMSE in nighttime comparisons [205].

The literature reports a broad accuracy range for land surface temperature retrievals from passive microwave radiometry, depending on sensor, algorithm, and surface/atmospheric conditions. In Table 5 a comparison of reported metrics from representative peer-reviewed studies is reported.

### 4.3. Vegetation Optical Depth

Accurate soil moisture retrieval from passive microwave observations requires explicit correction for attenuation caused by vegetation. This necessity has led to the derivation of Vegetation Optical Depth (VOD) as a by-product of many soil moisture retrieval algorithms. VOD data have been operationally available since 2010 from SMOS Level-2 products [6] and since 2015 from SMAP products [143,215], with SMOS Level-3 VOD provided by Al Bitar et al. [216], as well as from earlier AMSR [217] and AMSR-2 [218,219,220] retrievals. Over the past decades, retrieving VOD from microwave radiometry has become an increasingly important scientific task, both as an input to soil moisture retrievals and as an independent biophysical variable with ecological relevance. Advances in canopy radiative transfer modeling—particularly refinements to the τ–ω framework—have improved the representation of vegetation scattering and attenuation, especially over forested landscapes [152,153,221]. While VOD is not a direct surface property, numerous studies have demonstrated its link to vegetation water content, biomass, and structural attributes, underscoring its value for terrestrial ecosystem monitoring.

VOD is currently retrieved across multiple frequency bands. At higher frequencies (e.g., X- and C-band from AMSR-E and AMSR-2) [222], the signal primarily reflects the upper canopy layer. At lower frequencies, particularly L-band (SMOS and SMAP), microwaves penetrate deeper, interacting with leaves, branches, and trunks. L-band retrievals also exhibit a higher dynamic range, making them more sensitive to biomass changes and more robust under moderate vegetation cover [93].

For C- to K-band retrievals (6.6–19.35 GHz), three major global datasets are widely used: the Land Parameter Retrieval Model (LPRM) [223], the Global Land Parameter Data Record [224], and the Vegetation Optical Depth Climate Archive (VODCA) [139]. In the case of L-VOD from SMOS and SMAP, retrieval is typically based on an iterative minimization of a cost function defined as the sum of squared, weighted differences between measured and modeled brightness temperatures over multiple incidence angles [6]. Retrieval accuracy depends strongly on proper initialization of parameters, which is particularly critical in dense vegetation.

The link between VOD and vegetation water content was established early on [90] and later extended to show that Leaf Area Index (LAI) could serve as a proxy for water content [13,225]. SMOS currently initializes L-VOD using the maximum annual LAI effectively capturing seasonal dynamics such as leaf flush and senescence [165]. More recent studies have demonstrated strong correlations between L-VOD and tree canopy height measured by LiDAR [226], opening the way for integrating structural information into retrievals.

With the advent of high-resolution spaceborne LiDAR missions such as GEDI and ICESat-2, new methods have been proposed using canopy height (RH100) and Plant Area Index (PAI) as initialization parameters for L-VOD retrievals across all latitudes [226,227]. This approach improves the representation of both stable structural traits and variable vegetation states, particularly in forested ecosystems. Furthermore, these LiDAR-informed initializations can improve simultaneous retrievals of soil moisture, VOD, and scattering albedo (ω), which was tested in Vittucci et al. [228], where a three-parameter algorithm retrieved soil moisture, τ, and ω concurrently for all SMOS forest pixels. Similar methods have been successfully applied in dense tropical forests such as the Amazon and Congo basins [229], demonstrating their potential to reduce uncertainties in highly vegetated environments.

Recent developments also point toward multi-sensor VOD retrievals that combine L-band radiometry with radar backscatter, LiDAR structural metrics, and hyperspectral vegetation indices [230,231]. These integrated approaches not only improve the physical basis of VOD estimates but also open possibilities for monitoring vegetation stress, biomass changes, and carbon fluxes at unprecedented spatial and temporal scales.

Despite its potential and increasing use in ecological and hydrological studies, the application of VOD products is constrained by the near-total absence of systematic, direct ground measurements of VOD. In the absence of direct in situ VOD observations, validation efforts are typically forced to rely on indirect proxies. Among the other most significant proxies considered in the literature are aboveground biomass (AGB) and forest canopy height. Under the hypothesis that VOD is linked with vegetative water content (VWC) and the tree’s structure, intercomparison with AGB and forest canopy height as derived from independent satellite observations, also fused with ground data, is, in general, accepted as valid to evaluate the VOD products’ accuracy at a global scale. These aspects will be expanded in the next section. Some key challenges and known error sources complicate the retrieval and subsequent validation of VOD. A primary limitation to be mentioned is the coarse spatial resolution of current passive microwave VOD products, which varies significantly from approximately 25 km (e.g., higher-frequency sensors like AMSR-E/AMSR2) to around 40 km (e.g., L-band sensors like SMOS), which presents a fundamental mismatch when compared to localized ground data. Furthermore, the retrieval algorithms themselves introduce uncertainties. For instance, the presence of snow on the ground is a major uncertainty factor; if not properly accounted for in the radiative transfer model, it can lead to a substantial VOD overestimation of up to 30% [232,233].

Despite the difficulties in validating VOD since the issues in directly measuring such a parameter at ground, some ground-based methods are present in the literature. These include in situ measures of VWC, and measurements of plant water status (such as water potential and live fuel moisture content (LFMC).

Recently, some novel studies produced results based on GNSS measurements [234,235]. Such a new method for retrieving VOD could also help validate passive microwave, also considering the well-identified requirement to proceed with wider investigations with respect to surface data (and about the mitigation of the effects due to the spatial resolution of VOD products). Table 6 compiles reported accuracy metrics from studies validating satellite-derived VOD products against ground-based measurements, and an example of the usage of GNSS data in a pioneering study.

### 4.4. Vegetation Properties

Passive microwave observations provide a powerful means to investigate vegetation characteristics, particularly in forested ecosystems. Numerous studies have linked the attenuation of microwave signals by vegetation to the VOD, which represents the integrated effect of plant water content and structural properties [89]. Because microwave wavelengths can penetrate and interact with moderately dense canopies, depending on frequency, passive microwave remote sensing has been shown to provide valuable information on AGB [90,92,223,239], as well as on forest canopy height [90,166,227,228].

Recent work has highlighted the potential of L-band VOD to characterize structural vegetation properties through cross-comparisons with independent LiDAR missions. For example, Vittucci et al. [227,228] compared SMOS L2 L-VOD with vegetation structure derived from ICESat-2 [240] and GEDI [241], showing robust correlations with canopy height (RH100) and plant area index (PAI) across biomes and latitude bands. Correlation strengths varied seasonally, with the highest values found in tropical forests (R > 0.88) and weaker correlations in boreal regions during transitional seasons. These results emphasize the sensitivity of L-VOD to vegetation structure and water content under different climatic regimes.

Theoretical and empirical studies further confirm that VOD reflects both vegetation density (biomass) and water content, with canopy geometry strongly influencing the frequency dependence of attenuation [62]. Because it is less prone to saturation than optical indices such as NDVI (which saturates around 50–80 Mg ha^−1^), microwave VOD can capture biomass dynamics up to at least 350 Mg ha^−1^ [81,92,242]. Seasonal and interannual VOD variations have also been linked to ecosystem-scale plant water storage and leaf phenology. For instance, Tian et al. [243] found that L-VOD captured synchronous seasonal dynamics with leaf phenology in temperate and boreal forests, while in tropical forests plant water storage lagged canopy development by several months.

Beyond L-band, multi-decadal records of VOD retrieved from AMSR-E (in the timeframe 2002–2011) and AMSR-2 (in the timeframe 2012–present) at C- and X-band frequencies have been instrumental in extending vegetation monitoring back to the early 2000s. These records have been used to assess long-term vegetation water dynamics, biomass variability, and climate–vegetation interactions [244,245]. Efforts such as the Vegetation Optical Depth Climate Archive (VODCA) [139,234] merge AMSR/AMSR-2 with other passive microwave missions to provide harmonized, multi-frequency datasets. More recently, machine learning approaches have enabled the derivation of L-band–equivalent VOD products by transferring SMAP-based information into the AMSR era, effectively bridging frequency differences and extending the utility of the AMSR record [246].

VOD has also been linked to carbon cycle processes. Several studies demonstrate strong correlations between VOD and Gross Primary Production (GPP), highlighting its relevance for photosynthetic activity and forest functional traits [247,248]. Recent findings further show that VOD responds to ecosystem functional properties such as Water Use Efficiency (WUE) and Light Use Efficiency (LUE), thereby bridging structural and functional ecosystem monitoring [249]. New work has clarified that VOD sensitivity to biomass is frequency dependent: L-band tends to be more responsive to woody components, while higher frequencies emphasize leaf and canopy water status [250,251].

Importantly, VOD can also be used to estimate vegetation water storage, which is critical for understanding plant water stress and drought responses. Because microwave absorption is strongly influenced by water, changes in vegetation water status dominate short-term VOD variability [80,252]. In contrast, long-term trends primarily reflect biomass accumulation or loss [81,92]. This dual sensitivity complicates interpretation but also provides an opportunity to disentangle biomass and water dynamics by analyzing temporal scales and environmental context [253]. Despite the lack of a global large-scale vegetation water storage product, significant progress is being made in this direction [147,254].

Beyond vegetation optical depth (VOD), the use of raw microwave brightness temperature and polarization indices has provided valuable insights into vegetation structure and dynamics. Polarization differences (e.g., H–V or polarization ratio indices) are particularly sensitive to canopy water content, leaf orientation, and structural anisotropy, as vegetation alters the polarization state of the microwave signal through scattering and depolarization effects [206,255,256]. These indices have been exploited to detect vegetation growth stages, discriminate crop types, and assess canopy density, with L-band TB polarization differences showing strong links to aboveground biomass and canopy closure [78,256,257,258]. In forested ecosystems, polarization metrics complement VOD by enhancing sensitivity to canopy geometry and leaf water content, improving the characterization of phenology and drought responses [80,259]. Moreover, multi-frequency polarization indices from missions such as AMSR2 and SMAP enable consistent cross-scale vegetation monitoring by capturing both woody and leafy components, with C- and X-band indices more responsive to herbaceous vegetation and short-canopy crops. Emerging work demonstrates that integrating polarization indices with VOD in retrieval frameworks can reduce uncertainty in disentangling biomass and water storage dynamics, highlighting their potential for advancing ecosystem monitoring in the context of climate variability [260]. Table 7 compares representative metrics for correlations between VOD and vegetation properties.

### 4.5. Snow Cover and Ice Monitoring

One of the most extensively investigated applications of passive microwave remote sensing is the estimation of snow accumulation over land surfaces. Snow cover scatters and partially absorbs microwave radiation; in boreal forests, this challenge is further complicated by the interception of snow by the canopy [263]. Because microwave interactions are strongly influenced by snow microstructure, both active and passive systems can provide critical information about snowpack state variables [12]. However, retrieving snowpack properties often results in underdetermined systems (i.e., more unknowns than independent observations), since snow depth, density, layering, grain size, and liquid water content can all affect microwave signals in different, partially confounding ways.

Wet snow is particularly problematic. During spring melt or winter rain-on-snow events, liquid water strongly absorbs microwave radiation, greatly reducing the ability to sense snow depth or ground conditions beneath the snowpack [86]. This limitation makes accurate retrieval of the surface freeze–thaw state challenging, though snowmelt itself remains detectable even at coarse resolution [264]. High-frequency microwave bands (C- and X-band) are the most widely used for snow applications, as dry snow is nearly transparent at L-band, producing only weak brightness temperature signatures. Nevertheless, passive L-band observations can provide unique sensitivity to melt onset and liquid water content. Ground-based experiments with L-band radiometers have demonstrated feasibility, though retrievals are strongly influenced by soil moisture and wet snow conditions [265,266,267]. Theoretical work has also shown that L-band brightness temperatures can contain information on snow density [268,269]. Space-borne studies with SMOS have advanced this line of research. Early applications targeted melt detection on the Greenland ice sheet [136,270], but validation of snow density retrievals was lacking. A recent breakthrough by Gao et al. [270] demonstrated the first successful retrieval of snow density from satellite-based L-band data, enabled by coupling SMOS observations with land-surface model outputs. However, reliance on auxiliary data highlights the need for improved stand-alone retrieval algorithms.

Beyond snowpack properties, SMOS observations have also been exploited to investigate cryospheric dynamics in polar regions. Over Antarctica, SMOS-derived ice thickness variations were found to correlate with bedrock topography, and L-band observations proved sensitive to the vertical temperature profile of the ice sheet [271,272]. These studies underscored the potential of L-band data for continental-scale cryosphere monitoring, although residual systematic errors in SMOS radiometry (initially identified in sea surface salinity retrievals) still necessitate improved calibration and error characterization strategies [273,274].

Furthermore, retrieving snow properties from L-band radiometry during melt conditions remains a major open challenge [275]. The presence of liquid water within the snowpack drastically alters its dielectric properties, leading to a sharp increase in microwave emissivity and a loss of sensitivity to SWE [276]. Under these conditions, the L-band signal becomes dominated by surface melt layers, masking information from deeper snow layers and introducing significant retrieval uncertainties [268,277]. Moreover, the strong temporal variability of melting and refreezing processes produces non-stationary microwave signatures that are difficult to capture with current radiative transfer models.

More recently, novel approaches have further advanced microwave snow remote sensing. New retrieval algorithms using deep learning and data assimilation have improved the capacity to disentangle snow depth, density, and liquid water effects across scales [138,278,279]. Passive L-band studies have been integrated into hybrid modeling frameworks to better resolve snow water equivalent (SWE) in boreal and alpine regions [280]. At the same time, multi-sensor fusion efforts (e.g., combining SMOS, SMAP, AMSR2, and Sentinel missions) are demonstrating the ability to provide consistent, global snow datasets for climate studies [281,282]. Finally, recent climate-focused applications have highlighted the importance of microwave-based snow monitoring for quantifying freshwater resources and snow–albedo feedbacks in a warming climate [283,284].

Passive microwave remote sensing missions demonstrate variable accuracy in snow cover and ice monitoring dependent on sensor characteristics, retrieval algorithms, and environmental conditions. For snow depth and SWE retrievals at higher frequencies (10–89 GHz), root mean square errors (RMSE) typically range from 6.5 cm to 43 cm, with bias values spanning −2 cm to 24 cm, correlation coefficients (R) between 0.39 and 0.89 [284,285,286,287,288,289].

L-band sensors offer unique advantages including greater penetration into snow and thin sea ice (enabling thickness retrievals up to 0.5–1.0 m), sensitivity to snow density and thermal insulation effects, and reduced scattering losses in dry snow, but face limitations including reduced sensitivity to deep dry SWE due to low dielectric contrast, dependence on complex forward models and auxiliary data (with model-observation TB RMSE of 3–5 K). Furthermore, biases between sensors require careful intercalibration (SMAP-SMOS TB differences of 2.7–5 K before correction) [284,290,291,292,293,294]. In Table 8 a comparison of reported metrics from representative peer-reviewed studies is reported.

### 4.6. Surface Freeze–Thaw State

Based on the strong dielectric contrast between water and ice at microwave frequencies, brightness temperature (TB) measurements are widely used to detect the surface freeze–thaw (F/T) state. Changes in the phase state of liquid water have a pronounced effect on soil emissivity, as the crystalline structure of frozen water causes a sharp reduction in soil dielectric constant (ε) and consequently alters the emission signature [295]. In freezing soils, this rapid decline in ε produces a clear increase in microwave emission from the surface, enabling F/T detection through passive microwave observations.

Operational approaches include threshold-based techniques [83,86] and temporal change detection algorithms [78,296,297,298,299], both of which exploit daily fluctuations in TB. During the transition between frozen and thawed states, the impact of polarization becomes critical: horizontal polarization is more sensitive than vertical polarization at oblique incidence angles, making the polarization ratio an effective discriminator of surface F/T dynamics [85,266,300].

Recent advances have increasingly leveraged machine learning and data fusion methods. Incorporating ensemble learning and dynamic selection algorithms has been shown to significantly improve F/T retrieval accuracy, providing new opportunities to monitor cryosphere–climate interactions [83,301,302,303,304,305]. Satellite-based passive microwave measurements, such as those from FY-3B, have demonstrated strong potential when combined with edge-detection and discriminant-function approaches, achieving reliable results in complex terrains such as the Qinghai–Tibetan Plateau [306].

At lower frequencies, notably the L-band, retrievals benefit from the strong dielectric contrast between frozen and thawed soils, while also minimizing attenuation effects from snow and vegetation [85,86]. However, forested regions remain a challenge: canopy cover reduces the permittivity contrast, while trees exhibit their own seasonal dielectric cycle, leading to phase lags in F/T detection compared to open soils [307]. This canopy effect underscores the importance of considering vegetation–soil interactions in F/T monitoring.

Both SMOS and SMAP currently provide operational F/T state products at daily resolution and with a latency of 1–2 days, making them valuable resources for climate and hydrological studies [85,308,309,310]. Recent work has highlighted the potential of integrating these L-band products with higher-frequency observations (e.g., AMSR2, Sentinel-1) to enhance retrieval robustness under diverse environmental conditions [311,312]. Moreover, advanced deep learning frameworks trained on multi-sensor datasets [313,314] have demonstrated improved generalization across biomes, including boreal forests and tundra.

Classification accuracies typically range from 78% to 92%, varying by sensor, orbit node, region, and land cover characteristics. Major limiting factors include coarse spatial resolution (9–36 km), vegetation attenuation, topographic complexity, mixed pixels, temporal sampling constraints, and algorithm-specific limitations [84,86,231,232,233,303,315,316]. In Table 9 a comparison of reported metrics from representative peer-reviewed studies is provided.

### 4.7. Flood Monitoring

Passive microwave radiometry has proven to be a valuable tool for flood monitoring, primarily due to its ability to provide frequent global observations with minimal sensitivity to cloud cover and atmospheric conditions [317,318]. Early pioneering studies demonstrated that TB measurements, particularly in the horizontal polarization at ~36–37 GHz, are highly sensitive to the presence of surface water because of the strong emissivity contrast between water and land [319,320]. Sensors such as AMSR-E, AMSR-2, SSM/I, and SSMIS have been extensively used to exploit this differential response, allowing the detection of expanding surface water bodies and river discharge dynamics. Increased river discharge is typically accompanied by both a higher stage and a larger inundated area, which can be tracked with passive microwave observations [321,322].

Algorithms that exploit emissivity differences between dry and inundated surfaces have further improved the ability to monitor flood extent and evolution. Such approaches enable comparisons with optical satellite products and hydrological measurements and are particularly effective when applied to C- and L-band TB data, where the longer wavelengths penetrate vegetation and allow flood detection even under moderately dense canopies [323]. This advantage makes microwave radiometry complementary to optical sensors, which are often hindered by cloud cover during flood events.

Despite the relative robustness of microwave radiometry to cloud contamination, rainfall introduces a notable source of uncertainty in the interpretation of TB signals [324]. At frequencies above ~18 GHz, precipitation-sized droplets and ice particles scatter and absorb microwave radiation, leading to both attenuation of the land–water emissivity contrast and spurious enhancements in brightness temperature [325,326]. This effect is especially pronounced at 36–89 GHz, where heavy rainfall can mask surface water signals or mimic flood signatures, complicating flood detection algorithms [327]. In addition, the presence of wet vegetation during and after rainfall events can alter surface emissivity, further reducing the contrast between inundated and non-inundated areas [328].

Several studies have addressed these challenges by incorporating rainfall screening and correction techniques into flood monitoring algorithms. For instance, blended radiometric products often integrate coincident precipitation estimates from active sensors such as the TRMM Precipitation Radar or GPM Dual-frequency Precipitation Radar to flag rain-contaminated observations [329,330]. At lower microwave frequencies (<10–19 GHz), the direct sensitivity to rainfall is weaker, making them more reliable under precipitating conditions, but at the cost of coarser spatial resolution [331]. Ongoing research is therefore exploring frequency-dependent corrections and radiative transfer modeling for explicitly accounting for rainfall effects to better distinguish true flood signatures from precipitation-induced anomalies [332,333].

Such studies also exploit previous research on the estimation of overland precipitations through TB [334,335,336] or microwave soil moisture data [337,338].

Other advanced methods integrating multi-sensor passive microwave data have been developed. For example, long-term flood monitoring systems such as the Global Flood Detection System (GFDS) have combined AMSR-E/AMSR-2 and SSMIS data to deliver near real-time flood detection at continental to global scales [320]. Other techniques that couple radiometry with hydrological and hydraulic models have significantly improved accuracy in estimating flood extent and dynamics [339,340,341].

Recent research has also leveraged machine learning and data assimilation approaches to better extract flood information from passive microwave TB observations [342,343,344]. Similarly, Prigent et al. [331] highlighted the potential of multi-frequency microwave radiometry to capture wetland and floodplain dynamics over tropical regions, a critical step toward linking floods with carbon and methane fluxes.

Looking forward, the synergy of next-generation microwave missions, such as CIMR (Copernicus Imaging Microwave Radiometer, expected in 2029), with SMOS, SMAP, AMSR-2, FY3B and FY3C is anticipated to significantly enhance soil moisture and flood monitoring capacity [345,346].

Passive microwave observations provide high temporal resolution (often daily or sub-daily), which is crucial during rapidly evolving flood events. However, its accuracy is typically constrained by the coarse spatial resolution. Furthermore, enhanced resolution products, such as those combining data from the NASA SMAP sensor, have shown high performance in estimating Fractional Water (FW) cover, with advanced models like LSTM achieving a high Area Under the Curve (AUC) value of 0.93 and a general mapping accuracy of ~0.9 when validated against Sentinel-1 SAR images in favourable conditions (flat regions with low vegetation) [346]. In Table 10 a comparison of reported metrics from representative peer-reviewed studies is provided.

In conclusion, passive microwave radiometry remains one of the most effective tools for flood monitoring at large scales. Its all-weather capability, long time series, and sensitivity to open water dynamics make it a cornerstone for operational and research applications, increasingly supported by machine learning, data fusion, and hydrological model integration.

## 5. Data Assimilation Methodologies for Passive Microwave Remote Sensing

Passive microwave remote sensing has become an indispensable component of Earth system monitoring, providing continuous and all-weather observations of surface and subsurface hydrological variables. Several key satellite missions have contributed significantly to this domain by providing global measurements of brightness temperature across multiple frequencies and polarizations. These data underpin retrievals of soil moisture, snow water equivalent and vegetation optical depth, among other variables.

The assimilation of these data into Earth System Models (ESMs) has evolved rapidly over the past decade, leading to both operational and research-grade implementations. This section reviews the principal methodologies used to assimilate PMW observations, with emphasis on their theoretical foundations, representative applications, and demonstrated performance.

The SMOS mission offers L-band (1.4 GHz) multi-angle Tb observations with high sensitivity to surface soil moisture and moderate vegetation cover, facilitating both direct Tb assimilation and Level-2 retrieval assimilation into land surface models [351,352]. Similarly, SMAP, with its consistent calibration and near-daily global coverage, serves as the foundation of NASA’s operational Level-4 Soil Moisture (L4_SM) product, which assimilates Tb using the GEOS-5 ensemble-based system at 9 km spatial resolution [214,353]. AMSR-2, operating in C-, X-, and K-bands, complements L-band missions by enhancing sensitivity to vegetation water content and snowpack conditions [354,355]. Long-term multi-frequency observations from SSM/I and SSMIS provide crucial data for freeze–thaw detection and climate reanalyses, particularly in regional systems such as the China Land Data Assimilation System (CLDAS) [214].

### 5.1. Data Assimilation Methodologies Applied to Passive Microwave Remote Sensing Data

Data assimilation techniques combine satellite observations with model forecasts to generate optimal estimates of geophysical states. In the context of passive microwave remote sensing, these methods must account for nonlinear radiative transfer (RT) relationships, observation biases, and the high dimensionality of Earth system models. A comparative synthesis of the principal DA methodologies for passive microwave observations is presented in Table 11, highlighting their operational readiness, computational demand, and performance characteristics. The key aspects of the main DA methodologies are provided in the following paragraphs.

#### 5.1.1. Ensemble Kalman Filter and Variants

The Ensemble Kalman Filter (EnKF) is the most extensively used DA approach for PMW observations. As a sequential, ensemble-based Monte Carlo algorithm, it propagates an ensemble of model states forward in time and updates them based on observed Tb or derived retrievals. The Local Ensemble Transform Kalman Filter (LETKF) applies localization to mitigate spurious correlations and enhance scalability in high-dimensional land surface systems. EnKF and LETKF techniques have been successfully applied for assimilating SMOS and SMAP L-band Tb into land surface models such as GEOS-5, Noah-MP, and AWRA-L. The NASA SMAP Level-4 Soil Moisture (L4_SM) product employs an EnKF-based Tb assimilation framework to produce global, 9 km, 3-hourly soil moisture and root-zone estimates [351,353,356]. Similarly, AMSR-2 multi-frequency Tb assimilation using LETKF has improved SWE estimation in forested regions [351].

The EnKF’s ability to represent flow-dependent, spatio-temporal error covariances is a major strength, yet it assumes Gaussian error distributions and demands significant computational resources (typically 20–100 ensemble members). Reported performance metrics include unbiased RMSE ≈ 0.034 m^3^·m^−3^ for SMAP Tb assimilation in GEOS-5 [356], and improved soil moisture correlations from 0.54 to 0.77 with joint SMAP–SMOS assimilation in AWRA-L [357].

#### 5.1.2. Extended Kalman Filter (EKF)

The Extended Kalman Filter (EKF) linearizes both the system dynamics and the observation operator around the current state estimate. While computationally efficient, the EKF is less robust under strongly nonlinear conditions, such as those encountered in radiative transfer (RT) models for microwave emission. The European Centre for Medium-Range Weather Forecasts (ECMWF) employs an EKF to assimilate SMOS Tb into its H-TESSEL land surface model, improving soil moisture analyses particularly in data-sparse regions [358]. Despite its limitations, the EKF remains a practical option for global operational systems that require numerical stability and moderate computational cost.

#### 5.1.3. Particle Filters and Smoothers

Particle filters (PFs) constitute a class of sequential Monte Carlo methods that represent the posterior distribution using a weighted ensemble of particles. Each particle’s weight is updated according to the likelihood of observed Tb, allowing accurate treatment of non-Gaussian and nonlinear relationships. Particle smoothers extend this approach to temporal windows, enabling simultaneous estimation of states and model parameters.

PFs have been applied to assimilate AMSR-2 Tb into snowpack models such as Crocus, effectively handling the strong nonlinearities between Tb and SWE [355]. Particle smoothers using Sequential Importance Resampling (SIR) have also been applied to jointly estimate vegetation optical depth and surface roughness parameters from multi-frequency Tb time series [359,360].

These methods excel in modeling multimodal posterior distributions and jointly estimating states and parameters but are computationally demanding. Notably, AMSR-2 Tb assimilation with a PF reduced SWE bias by 68% (from 23.7 to 7.5 kg·m^−2^) and decreased relative percentage error from 19% to 14.1% [355]. PF-based smoothers have also produced temporally stable RT parameters, improving Tb simulation fidelity [360].

#### 5.1.4. Hybrid and Advanced Approaches

Systematic observation biases from instrument calibration, seasonal drifts, or model discrepancies can degrade assimilation outcomes. Dual-cycle or online bias correction frameworks address this by jointly estimating state and bias parameters within nested optimization loops. These systems dynamically update bias terms during assimilation, outperforming static climatological corrections. Studies report that online bias correction for SMAP Tb significantly enhances soil moisture retrieval consistency and reduces long-term drift [352]. Additional advanced methods are based on machine learning (ML) which offers new avenues for improving DA efficiency and resolution. Applications include:

1. Downscaling: Random forest and neural network models downscale coarse (∼36 km) PMW soil moisture to 5–10 km resolution using terrain, vegetation, and soil properties as predictors [361].
2. Observation operator emulation: ML models emulate RT relationships (e.g., LAI–VOD mapping), accelerating forward simulations without compromising accuracy [362].
3. Multi-mission fusion: ML algorithms integrate SMAP, SMOS, and AMSR-2 datasets into unified, bias-corrected products for assimilation [362].

These ML-enhanced systems achieve unbiased RMSE < 0.06 m^3^·m^−3^ in high-resolution soil moisture estimation [361], demonstrating their potential as scalable, hybrid frameworks for operational ESMs.

### 5.2. Applications in Earth System Models

The assimilation of passive microwave observations into Earth System Models (ESMs) has become a cornerstone in improving the representation of hydrological, cryosphere, and coupled land–atmosphere processes. Through the integration of satellite-derived Tb or soil moisture retrievals, DA systems correct model biases, constrain uncertainties, and enhance short- to medium-range predictions. Among the main components of ESMs, the most mature PMW DA applications are found in land surface models (LSMs), followed by cryosphere models and coupled land–atmosphere frameworks.

Overall, the integration of passive microwave data into Earth system modeling frameworks has demonstrated significant benefits across a range of environmental domains. Ensemble-based techniques, such as the EnKF, have become operational standards for LSMs, while nonlinear particle filter methods have proven effective for snow and ice modeling. More recently, coupled land–atmosphere assimilation has emerged as a promising avenue for improving surface–atmosphere feedback representation. A comparative synthesis of the principal applications of passive microwave DA in ESMs is presented in Table 12, while details on the main outcomes are provided in the following paragraphs.

#### 5.2.1. Land Surface Models

Land surface models have served as the principal domain for PMW data assimilation, owing to the strong sensitivity of microwave emissions to surface and subsurface soil moisture, vegetation, and snow conditions. The assimilation of Tb or derived retrievals enables LSMs to adjust key hydrological and thermodynamic states, improving estimates of surface soil moisture, root-zone soil moisture, snow water equivalent (SWE), vegetation optical depth (VOD), and freeze–thaw dynamics. One of the most prominent examples is the NASA GEOS-5 Catchment Land Surface Model (CLSM), which employs an Ensemble Kalman Filter (EnKF) to assimilate SMAP and SMOS Tb observations. This configuration forms the basis of the SMAP Level-4 Soil Moisture (L4_SM) operational product, delivering 9 km, 3-hourly global soil moisture estimates validated against extensive in situ and independent remote-sensing datasets [351,352,356]. Similarly, the European Centre for Medium-Range Weather Forecasts (ECMWF) integrates SMOS Tb using an Extended Kalman Filter (EKF) within its H-TESSEL land surface scheme, which directly improves modelled soil moisture and surface energy fluxes used in numerical weather prediction [358].

In the United States, the NOAA/NASA Land Information System (LIS) provides a modular DA framework that supports the assimilation of SMOS and SMAP retrievals into several LSMs, such as Noah, Noah-MP, and VIC, thereby enhancing hydrological and agricultural forecasts [363]. Similarly, the Australian Water Resources Assessment Landscape (AWRA-L) model operationally assimilates SMAP and SMOS data to improve large-scale water balance predictions, substantially increasing drought monitoring accuracy across the Australian continent [357,364]. The China Land Data Assimilation System (CLDAS) extends these efforts regionally by incorporating SSM/I and AMSR-2 Tb observations for soil moisture and freeze–thaw estimation over East Asia [354]. Across these systems, the assimilation of passive microwave data yields measurable benefits. Reported outcomes include soil moisture correlation improvements of +0.10 to +0.26, unbiased RMSE reductions of 0.002–0.008 m^3^·m^−3^, and SWE bias reductions reaching 68% in forested regions [351]. These consistent gains highlight the robustness of PMW DA in constraining land surface hydrology and improving the reliability of operational LSM outputs.

#### 5.2.2. Cryosphere Models

The cryosphere benefits substantially from PMW data assimilation, as microwave signals are particularly sensitive to variations in snow grain size, density, and liquid water content. Assimilation of multi-frequency Tb from AMSR-2 and SSM/I improves the characterization of snow and sea ice, which are critical for cold-region hydrology and climate feedback. A notable implementation is found in the Crocus snowpack model, where a particle filter framework assimilates AMSR-2 Tb to estimate SWE [356]. This nonlinear approach accommodates the strong non-Gaussian relationship between snow properties and Tb, outperforming linear assimilation techniques. Similarly, the AWI Climate Model, developed by the Alfred Wegener Institute, applies a coupled sea ice DA system that incorporates PMW data for improved estimates of sea ice concentration and thickness, supporting seamless prediction in polar regions [365].

Performance metrics from these systems demonstrate significant gains: for example, in the Crocus model, the assimilation of AMSR-2 Tb reduced SWE bias by 68% (from 23.7 to 7.5 kg·m^−2^) and decreased the relative percentage error from 19% to 14.1% [356]. These findings underscore the capability of particle filter-based assimilation to manage nonlinear and multimodal state relationships, thereby enhancing the realism of snow and ice process simulations.

#### 5.2.3. Coupled Land–Atmosphere Systems

Recent advances have extended passive microwave data assimilation toward coupled land–atmosphere systems, enabling two-way interactions between surface and atmospheric states. Such frameworks exploit the strong influence of land hydrology on boundary-layer thermodynamics, offering an integrated perspective on water and energy exchanges. The GEOS Land–Atmosphere Data Assimilation System (LADAS) exemplifies this approach. By assimilating SMAP Tb observations into the coupled GEOS-5 model, LADAS simultaneously updates soil moisture, near-surface air temperature (T2m), specific humidity (q2m), and surface heat fluxes [354]. This coupling allows the surface information to propagate vertically, improving the consistency between land and atmospheric analyses. Evaluation results reveal reductions in T2m maximum RMSE by 0.04 K, q2m RMSE by 0.05 g·kg^−1^, and observation-minus-forecast Tb residuals by up to 0.1 K [354]. These modest yet systematic improvements enhance both the accuracy and physical coherence of coupled model forecasts.

### 5.3. Key Challenges, Limitations, and Recent Advances in Passive Microwave Data Assimilation

Passive microwave DA has matured into a critical tool for improving land, cryosphere, and coupled Earth system predictions. Despite substantial progress, several technical and computational challenges remain, which have motivated methodological innovations that have improved both performance and applicability.

One of the primary technical challenges in PMW DA is the presence of systematic observation biases. These biases arise from instrument calibration drift, seasonal variability, and inter-sensor differences. Without proper correction, they propagate into the assimilated model states, leading to systematic errors. Recent studies have demonstrated that online bias correction schemes, such as dual-cycle DA systems, outperform static climatological corrections by dynamically estimating observation biases during the assimilation process [269,351].

Another challenge is radiative transfer (RT) model uncertainty, which arises when forward simulating Tb from model states. RT models depend on parameters such as vegetation optical depth, surface roughness, and soil texture, which are often uncertain. Errors in these parameters directly affect DA updates. Recent approaches using particle smoothers or dual-cycle schemes allow joint estimation of states and RT parameters, stabilizing forward-model simulations [360,361].

Spatial resolution mismatch is also a key limitation. PMW observations typically have footprints of tens of kilometres, while land and coupled Earth system models often operate at 1–10 km resolution. Specialized observation operators, such as spatial averaging or super-observation techniques, are necessary to prevent smoothing of fine-scale model features [4]. Additionally, vegetation and snow effects reduce the penetration of microwave signals in dense forests or deep snow, degrading retrieval accuracy. Advanced RT models that explicitly represent vegetation attenuation and snow scattering are required to enable effective DA in these challenging regions [355,356].

Finally, the choice of assimilation variable affects DA performance. Direct assimilation of brightness temperatures often outperforms the assimilation of Level-2 soil moisture retrievals because it preserves the full information content and avoids biases introduced by retrieval algorithms. However, this requires accurate RT operators and robust bias correction [358,363].

Computational burden is another limiting factor. Ensemble-based methods such as the EnKF and LETKF require running multiple forward simulations (typically 20–100 ensemble members) and evaluating RT operators for each Tb observation. Particle filters, while capable of handling non-Gaussian and strongly nonlinear processes, require hundreds to thousands of particles to avoid degeneracy, making them computationally very expensive [356,360]. Operational latency is also a concern, particularly for near-real-time products such as SMAP L4_SM, which requires rapid data ingest, quality control, assimilation, and dissemination [366]. Joint state-and-parameter estimation further increases computational cost due to the expanded dimensionality of the assimilation problem [360,361].

To address the challenges, the last decade has seen significant methodological and technological advances in passive microwave DA, leading to improved accuracy, efficiency, and new applications across the Earth system.

Dynamic bias correction within the assimilation cycle has become increasingly adopted. Dual-cycle assimilation systems estimate observation biases concurrently with model states, improving consistency and reducing systematic errors. SMAP-based studies illustrate that online bias correction outperforms static schemes, leading to more accurate soil moisture products [1,12].

Machine learning (ML) techniques have enhanced DA workflows in several ways. ML models are applied to downscale coarse PMW soil moisture observations from 36 km to finer resolutions (5–10 km) using ancillary predictors such as topography and vegetation indices [362,363]. ML also serves as a surrogate observation operator, emulating computationally expensive RT models for fast forward simulations, and enables multi-mission fusion to integrate SMAP, SMOS, and AMSR-2 observations into unified DA inputs [365]. ML-enhanced systems have demonstrated unbiased RMSE below 0.06 m^3^/m^3^ for high-resolution soil moisture [362].

An additional factor for future improvements is that the joint assimilation of L-band (SMOS, SMAP) and higher-frequency (AMSR-2) data improves temporal coverage and exploits complementary sensitivities. For instance, merging SMAP and SMOS observations increases availability by 41% and improves soil moisture correlation from 0.54 to 0.77 [358]. Similarly, combining soil moisture and vegetation optical depth (VOD) assimilation enhances both hydrologic states and vegetation flux estimates [361].

Other improvements have been achieved on the RT side. Advanced RT-based operators now support multi-angle and multi-polarization simulations, passive–active sensor integration, and specialized operators for model–observation resolution mismatch. These innovations improve the realism of forward simulations, reduce biases, and enable the assimilation of complex datasets such as radiometer and SAR observations [353,354].

Dual state–parameter estimation and temporal particle smoothing have improved the stability of RT model parameters, such as vegetation optical depth and surface roughness, reducing systematic forward-model errors and improving assimilation consistency [360,361]. Finally, also coupled Land–Atmosphere Data Assimilation methods benefited by improvements such as weakly coupled DA systems which have demonstrated that assimilating passive microwave observations into coupled land–atmosphere models improves both terrestrial and atmospheric variables. For example, SMAP Tb assimilation into GEOS LADAS reduced the RMSE of near-surface temperature (T2M) by 0.04 K and specific humidity (Q2M) by 0.05 g/kg, while improving soil moisture estimates [352].

#### Future Directions

Looking ahead, next-generation missions such as CIMR and ROSE-L will provide multi-frequency, high-resolution PMW observations, enabling enhanced multi-sensor assimilation. Methodological advances, including deep learning-based RT operators, hybrid ensemble-variational DA (e.g., 4D-EnVar), multi-scale assimilation frameworks, and improved uncertainty quantification, are expected to further enhance DA fidelity. Expanding applications include fully coupled Earth system DA, carbon cycle monitoring, operational hydrological forecasting, and multi-decadal climate reanalyses integrating SSM/I, AMSR-E, AMSR-2, SMOS, and SMAP datasets. Together, these advances promise significant improvements in both the accuracy and scope of Earth system prediction.

## 6. Future of Satellite Microwave Radiometry for Land Monitoring

Several upcoming and proposed satellite missions are poised to significantly advance Earth observation capabilities through state-of-the-art microwave radiometry, extending the legacy of missions such as SMOS, SMAP, and AMSR-E/2. These missions aim to improve monitoring of key components of the water, energy, and carbon cycles while supporting climate services and policy frameworks.

### 6.1. Copernicus Imaging Microwave Radiometer (CIMR)

The CIMR mission, part of the Copernicus Sentinel Expansion, is designed to deliver daily, all-weather observations for both polar and global applications. Equipped with a wide-swath, conically scanning multi-frequency radiometer operating across L-, C-, X-, K-, and Ka-bands, CIMR will measure key environmental variables such as sea-surface temperature, sea-ice concentration and thickness, sea-surface salinity, snow parameters, soil moisture, vegetation properties, and precipitation [367,368,369]. Covering approximately 95% of the globe each day, with sub-daily Arctic revisit capability and high spatial resolution (~5 km for sea ice), CIMR is scheduled for sequential launches—CIMR-A around 2029 and CIMR-B around 2035—to ensure more than 15 years of observation continuity [369]. The mission will support Arctic policy, climate monitoring, and Copernicus services, with open and timely data delivery within hours of sensing [369].

### 6.2. Copernicus Polar Ice and Snow Topography Altimeter

The Copernicus Polar Ice and Snow Topography Altimeter (CRISTAL) mission will carry a dual-frequency radar altimeter and a microwave radiometer. The latter will provide measurements of snow depth overlying sea ice, a key parameter for accurate retrieval of ice thickness from altimetry data. CRISTAL is expected to play a pivotal role in monitoring ice sheets, glaciers, and global sea-level rise [370].

The microwave radiometer on CRISTAL, known as the Advanced Microwave Radiometer for CRISTAL (AMR-CR), is designed to provide critical atmospheric corrections for the primary radar altimeter. Specifically, it will measure the integrated water vapor (IWV) content in the atmosphere, as atmospheric water vapor causes a delay in the radar signal, which, if uncorrected, would introduce significant errors into the altimeter’s height measurements [371]. The AMR-CR operates at three key frequencies: 18.7, 23.8, and 34 GHz. These frequencies are carefully selected to provide optimal sensitivity to water vapor and other atmospheric constituents. The 23.8 GHz channel is particularly sensitive to water vapor, while the other channels help to separate the signal from other sources like liquid water and surface emissions.

### 6.3. Advanced Microwave Scanning Radiometer Third Generation

The Japanese mission GOSAT-GW (Global Observing Satellite for Greenhouse gases and Water cycle) will be equipped with AMSR-3, the next-generation Advanced Microwave Scanning Radiometer. AMSR-3 will extend the heritage of AMSR-E and AMSR-2, with enhanced frequency coverage for improved snow detection, water vapor profiling, and surface monitoring over land and oceans. The AMSR-3 instrument will represent a significant technological advancement, operating with a large, 2.0 m diameter offset parabolic antenna to achieve a wide swath of over 1530 km [372].

A key enhancement of AMSR-3 is its expanded frequency set, which includes new high-frequency channels at 165.5 GHz and a pair of channels at 183.31 GHz [373]. These channels are specifically designed to improve the retrieval of atmospheric water vapor profiles and solid precipitation, such as snowfall, which are difficult to measure with lower-frequency channels due to their insensitivity to ice and small-scale atmospheric features. The instrument maintains the dual-polarization capability (horizontal and vertical) across most of its channels, which is crucial for distinguishing between various surface types and atmospheric conditions [372,373]. The spatial resolution of AMSR-3 varies with frequency, ranging from approximately 3 km × 5 km at 89.0 GHz to 34 km × 58 km at the lowest frequencies (6.925 and 7.3 GHz), providing a balance between fine-scale detail and broad-area coverage [372,373]. The instrument employs a conical scanning geometry at a fixed incidence angle of 55°, ensuring consistent spatial resolution and radiometric quality across the entire swath.

### 6.4. Cryospheric Radiometer Mission Concept

The Cryospheric Radiometer Mission Concept (CryoRad) mission is a visionary Earth Explorer 12 candidate proposed by ESA—a single satellite mission centered on a broadband low-frequency microwave radiometer operating across 0.4 to 2 GHz [374]. The mission has been retained for further assessment in the initial Phase-0. The payload is based on a radiometer designed to penetrate deep into ice and cold ocean waters, enabling first-ever spaceborne observations of ice-sheet temperature profiles, sea-ice thickness, and sea-surface salinity with significantly improved sensitivity over existing L-band instruments [318]. Three principal science objectives define CryoRad’s potential impact:

1. Ice-Sheet Stability: CryoRad will provide temperature profiles from the surface to the base of Greenland and Antarctic ice sheets, filling critical observational gaps beyond sparse boreholes and models [375].
2. Polar Freshwater Cycle: By halving uncertainties in sea-surface salinity compared to current L-band missions, CryoRad will improve knowledge of high-latitude hydrology and density-driven ocean circulation.
3. Sea-Ice Dynamics: CryoRad will measure sea-ice thickness (0.5–1 m) and salinity distribution—parameters never retrieved from space—advancing the monitoring of freshwater fluxes and polar stratification [375,376].

Key instrument features include nadir observations with circular polarization to mitigate Faraday rotation, continuous frequency scanning, and a swath of approximately 120 km, delivering ground resolutions from 45 km at 0.4 GHz down to 8 km at 2 GHz, with repeat times of ~3 days at high latitudes and ~10 days at the equator [375,376]. Originally proposed under the Earth Explorer 12 program, CryoRad has been commended for its scientific maturity, and studies are ongoing to refine its design and system requirements after the Phase-0 [374].

### 6.5. Fine-Resolution Explorer for Salinity, Carbon, and Hydrology

Proposed under ESA’s call for the Earth Explorer 12 program, Fine-Resolution Explorer for Salinity, Carbon, and Hydrology (FRESCH) represents a novel L-band aperture synthesis imaging radiometer designed to deliver high-resolution (~10–15 km) observations at the ocean–land–ice interface [377,378]. Unlike SMOS, FRESCH would rely on digital beamforming, allowing efficient signal processing from a large antenna array. Following algorithmic refinements to optimize computational demands, the mission concept now foresees a 172-antenna cross-rotated array capable of multi-polarization, multi-incidence angle imaging. FRESCH is expected to provide data for several Essential Climate Variables (ECVs), including sea surface salinity, soil moisture, biomass, and sea ice, while also contributing to dissolved carbon fluxes, permafrost monitoring, and cryosphere change assessments [378].

### 6.6. Sea-Air-Ice-Land INteractions

The Sea-Air-Ice-Land INteractions (SAILIN) mission was proposed under the recent ESA call for Earth Explorer-12 to study the rapid, small-scale dynamics of ocean surfaces, particularly in coastal, shelf, and marginal ice zones. The mission’s primary objective is to understand how these fast-evolving, sub-10 km ocean features mediate the exchange of key elements like heat, carbon, and nutrients across critical interfaces of the Earth System. SAILIN would utilize innovative technology on a single satellite to provide high-resolution, daily observations of surface currents, winds, and waves, which would be crucial for improving our understanding of air–sea fluxes, ocean carbon cycling, and the processes that affect sea ice growth and decay, filling a gap left by existing missions.

The proposed synergy of payloads would be based on the Tri-HEX concept [379], a novel 3-platform formation flying in general circular orbits, yielding an alias-free imaging system by means of high-resolution distributed L-band synthetic aperture radiometers combined with 3 GNSS-R instruments for enhancing the sensing capability of the sea roughness and the soil moisture in densely vegetated areas. With a 3 m envelope diameter, the three hexagonal spacecrafts fit within a Vega-C launcher’s usable envelope. It is anticipated that the effective radiometric sensitivity will be 0.2 K, surpassing figures linked to prior L-band missions (0.36 K and 0.3 K for SMOS and CIMR, respectively). In addition to increasing previous values (33–50 km for SMOS) and on the next scheduled L-band missions (36 × 64 km for CIMR), the spatial resolution at nadir is 15 km [379]. With a fully alias-free swath of 1200 km and an incidence angle range of 0–60 degrees, SAILIN offers multi-angular observational capabilities [379].

## 7. Conclusions

Microwave radiometry has established itself as a cornerstone in the global climate observing system, providing unparalleled capabilities for the continuous monitoring of ECVs. Over the past decades, passive microwave observations have generated long-term, harmonized records of soil moisture, snow cover, vegetation structure, and freeze–thaw dynamics, which are indispensable for understanding land–atmosphere interactions and the functioning of the global carbon and water cycles. The integration of these datasets into reanalyses and Earth system models has already improved the representation of hydrological and biogeophysical processes, thereby enhancing both short-term forecasting skill and the reliability of long-term climate projections.

Recent progress has underscored the potential of microwave observations to bridge temporal and spatial scales. VOD retrievals, derived from L-band sensors such as SMOS and SMAP as well as higher-frequency missions including AMSR-E and AMSR-2, now provide multi-decadal datasets for monitoring ecosystem functioning, biomass variability, and vegetation–climate feedback. Advances in dataset harmonization, frequency translation using artificial intelligence, and synergies with complementary observations such as LiDAR are positioning VOD as a key diagnostic for assessing terrestrial ecosystem resilience under climate stressors.

At the same time, passive microwave techniques have become increasingly important for monitoring the cryosphere. Snow emission modeling, despite persistent challenges under wet snow conditions and forested environments, has advanced to the point where multi-frequency retrievals can provide valuable insights into snow water equivalent and snowpack dynamics. Such observations, when combined with satellite missions focused on sea-ice and ice-sheet processes, offer a powerful means of quantifying freshwater fluxes and energy exchanges in the polar regions—areas that remain highly sensitive to climate change.

Despite the remarkable progress achieved in recent years, several challenges remain before the full potential of microwave radiometry can be realized. Retrievals under dense vegetation canopies, wetlands, and mountainous terrains continue to suffer from uncertainties, particularly when signal contributions from soil, snow, and vegetation overlap. Similarly, wet snow conditions and melting events still pose significant limitations to snow water equivalent retrievals, underscoring the need for advanced radiative transfer modeling and synergies with active microwave and optical observations. Another open challenge is the consistent cross-calibration of sensors across multiple missions, which is crucial for maintaining the continuity and accuracy of multi-decadal climate data records.

Advances in data assimilation, machine learning, and uncertainty quantification will further enable the exploitation of multi-frequency, multi-mission datasets within coupled Earth system models.

Looking ahead, the upcoming generation of missions, such as CIMR, AMSR-3 and CRISTAL (including potential evolutions of the proposed missions such as FRESCH, SAILIN and CryoRad), promises to greatly expand observational capability through enhanced spectral coverage, higher spatial resolution, and innovative antenna technologies. These missions will not only ensure continuity of critical ECV records but also open new avenues for quantifying processes at the land–ocean–ice interface. The assimilation of such data into coupled land, ocean, and climate models will further reduce uncertainties in Earth system projections, reinforcing the role of passive microwave radiometry in global climate monitoring and policy support.

In conclusion, microwave radiometry has evolved from a niche observational technique to a central pillar of Earth observation science. Its ability to provide all-weather, day-and-night observations of water, energy, and carbon dynamics makes it uniquely suited to capture the fingerprints of climate variability and change. Continued investment in sensor development, data harmonization, and model integration is essential to fully exploit its potential, ensuring that the scientific community can meet the growing societal demand for reliable climate information in the decades to come.

## Figures and Tables

**Table 1:** IEEE bands designation and their main applications in remote sensing (land surface applications are highlighted in italics).

IEEE Radar Band	Frequency Interval (GHz)	Remote Sensing Applications
P	0.3–0.5	*Soil moisture estimation also under forests*
		*Snow water equivalent*
		*Forest biomass and height*
L	1–2	*Soil moisture estimation also under forests*
		*Hydrology*
		*Vegetation and crop monitoring*
		*Vegetation biomass estimation*
		*Snow and ice monitoring*
		Sea ice concentration and ice sheet volume
		Ocean surface salinity and temperature
S	2–4	*Snow and ice monitoring*
		*Soil moisture estimation*
		Atmospheric sensing of clouds and precipitation
		Sea surface temperature
		Wind speed over ocean
C	4–8	*Soil moisture estimation*
		*Vegetation and crop monitoring*
		*Snowpack analysis*
		Rainfall estimation
X	8–12	*Snow and frost detection*
		Liquid water content of clouds
		Atmospheric temperature profiles
Ku	12–18	*Snow and ice properties*
		Rainfall estimation
		Ocean wind speed
K	18–26.5	*Snow thickness*
		*Soil moisture estimation*
		Rainfall estimation
		Atmospheric vertical temperature and humidity profiles
Ka	26.5–40	*High-resolution snow*, *ice properties*
		Precipitation, fog and cloud properties
V	40–75	Atmospheric temperature profiles
W	75–110	Liquid and frozen precipitation
mm ^1^	110–300	Snowfall and ice particles
		Water vapor profiles

^1^ The “mm” band is included as it extends the microwave radiometry range up to 300 GHz.

**Table 2:** Main passive radiometry satellite missions for land surface monitoring (land surface variables are highlighted in italic).

Mission Name	Space Agency/Partnership	Operating Bands (Frequency GHz)	Launch Year	Operational Status	Primary Applications
SMOS (Soil Moisture and Ocean Salinity)	European Space Agency (ESA)	L-band (1.4)	2009	Operational (extended)	*Soil Moisture*, Sea Surface Salinity
SMAP (Soil Moisture Active Passive)	National Aeronautics and Space Administration (NASA)	L-band (1.4)	2015	Operational (extended)	*Soil Moisture*, *Freeze/Thaw State*
Aquarius	NASA/Comisión Nacional de Actividades Espaciales (CONAE)	L-band (1.4)	2011	Ended 2015	*Soil Moisture*, Sea Surface Salinity
AMSR (Advanced Microwave Scanning Radiometer)	Japan Aerospace Exploration Agency (JAXA)	C (6.9 and 7.3), X (10.7), K (18.7 and 23.8), Ka (36.5), V (50.0), W (89.0)	2002	Ended 2011	*Soil Moisture*, Sea Surface Temperature, *Polar Region Monitoring*, *Fire Severity*, *Cryosphere*, Ocean Wind Speeds, Drought
AMSR-E (Advanced Microwave Scanning Radiometer for EOS)	JAXA/NASA	C (6.925), X (10.65), K (18.7 and 23.8), Ka (36.5), W (89.0)	2002	Ended 2011	*Soil Moisture*, Sea Surface Temperature, *Polar Region Monitoring*, *Fire Severity*, *Cryosphere*, Ocean Wind Speeds, *Drought*
AMSR2 (Advanced Microwave Scanning Radiometer 2)	JAXA	C (6.925 and 7.3), X (10.7), K (18.7 and 23.8), Ka (36.5), W (89.0)	2012	Operational	Hurricane Intensity, Global Precipitation, Snowfall, *Cryosphere*, Ocean Wind Speeds, Sea Surface Temperature, *Soil Moisture*, *Drought*
TRMM Microwave Imager (TMI)	NASA/JAXA	X (10.7), K (19.0 and 22.0), Ka (37.0), W (85.5)	1997	Decommissioned 2015	Rain Structure, Hurricane Intensity, Precipitation, *Flood Forecasting*, *Soil Moisture*, Ocean Wind Speeds, Sea Surface Temperature
GMI (GPM Microwave Imager)	NASA/JAXA	X (10.65), K (18.7), K (23.8), Ka (36.5), W (89.0), mm (166.0, 183 ± 3 and 183 ± 7)	2014	Operational (extended)	Tropical Cyclone Forecasting, Global Precipitation, Hurricane Forecasts, Ocean Wind Speeds, Sea Surface Temperature, *Soil Moisture*
SSM/I (Special Sensor Microwave/Imager)	U.S. Defense Meteorological Satellite Program (DMSP)	K (19.3), Ka (36.5 and 37.0), W (85.5)	1987 (first unit F8)	Data archive continually updated (various units decommissioned over time)	Hurricane Intensity, *Flood Detection*, *Soil Moisture*, Ocean Wind Speeds, Sea Ice
SSMIS (Special Sensor Microwave Imager/Sounder)	U.S. DMSP	Ka (37.0), W (91.7)	2003 (first unit F16)	Operational (some units still active, data flow until at least 2025/2026)	Tropical Cyclone Precipitation, *Flood Detection*, Snowfall, Ocean Wind Speeds, Sea Ice, *Soil Moisture*
SMMR (Scanning Multichannel Microwave Radiometer)	NASA	C (6.6), X (10.7), Ku (18.0), K (21.0), Ka (37.0)	1978	Ended 1987 (Nimbus 7 SMMR)	Sea Surface Temperature, Ocean Wind Speeds, *Flood Detection*, *Soil Moisture*, Sea Ice
Coriolis WindSat	U.S. Department of Defense (DoD)	C (6.8), X (10.7), Ku (18.7), K (23.8), Ka (37.0)	2003	Ended 2020	Ocean Surface Wind Speed, NWP Improvement, *Soil Moisture*
MHS instrument (on MetOp-A)	European Organisation for the Exploitation of Meteorological Satellites (EUMETSAT)/National Oceanic and Atmospheric Administration (NOAA)	W (89), mm (157.0, 183.31 ± 1, 183.31 ± 3 and 190.311)	2006	Ended 2021	*Surface Temperature*, Atmospheric Humidity Profiles, Precipitation Rate, Emissivity, Low Altitude Cloud Detection
MHS instrument (on MetOp-B)			2012	Operational (until 2027)	
MHS instrument (on MetOp-C)			2019	Operational (until 2027)	
MHS instrument (on NOAA-18)			2005	2017	
MHS instrument (on NOAA-19)			2009	Operational (until 2025)	
Microwave Imager (MWRI) on Fengyun-3 (FY-3) satellite series		10.65, 18.7, 23.8, 36.5, and 89.0 (GHz), each with dual polarization (Horizontal and Vertical)	2008 (FY-3A) 2010 (FY-3B), 2013 (FY-3C), 2017 (FY-3D), 2023 (FY-3F)	Operational Operational Operational	*Soil Moisture*, *Snow Cover*, Precipitation (rain rate), Cloud Liquid Water, Total Precipitable Water, Sea Surface Temperature, Sea Ice Concentration

**Table 3:** Comparison of RT Modeling Approaches for Vegetation, Snow, and Ice.

Medium	Primary Scatterers	Dielectric Properties	Dominant Processes	Typical Frequency Range	Main Modeling Approaches	Key Challenges
Vegetation (Forests, Crops)	Leaves, stems, trunks, branches	Moderate permittivity (~2–15, moisture-dependent)	Attenuation, volume scattering, double-bounce (trunks)	0.5–10 GHz (MIMICS, Tor Vergata model); L-band for SMOS/SMAP	τ–ω model, first-order RT, matrix doubling	Complex canopy geometry, orientation statistics, seasonal variation in water content
Snow (Seasonal Snowpacks)	Ice grains (spherical/ellipsoidal), air inclusions	Low permittivity (~1.2–1.9 dry snow; ↑ with liquid water)	Volume scattering (Mie), absorption (wet snow), ground emission transmission	1.4–37 GHz	MEMLS, DMRT, layered RT models	Accurate grain size/density profiles, liquid water fraction, stratigraphy
Glacial Ice/Polar Ice Sheets	Ice crystals, air bubbles, brine pockets	Higher permittivity (~3.15 for pure ice; varies with salinity)	Volume scattering, anisotropic propagation, absorption in wet layers	0.5–37 GHz (active & passive), radar altimetry	DMRT, anisotropic RT, layered ice models	Large-scale heterogeneity, anisotropy, coherent effects, melt layer detection

The ↑ symbol indicates an increase in metrics with respect to the state of the art.

**Table 4:** Representative accuracy of SM retrieval algorithms.

Mission	ubRMSE/RMSE	R	Representative Validation Notes and Source
SMAP (L-band only passive)	≈0.04–0.05 m^3^/m^3^ (radiometer SM product)	R ≈ 0.7–0.81	Validation vs. airborne and in situ (SMAPEx) found radiometer SM RMSE ≈ 0.05 m^3^/m^3^ and TB RMSE ≈ 3 K; global/core-site comparisons report ubRMSE ≈ 0.041 and R ≈ 0.81 [165,180]
SMOS (L-band)	ubRMSE ≈ 0.039–0.10 m^3^/m^3^ (product/version dependent)	R ≈ 0.4–0.80	SMOS IC product and later reprocessing approach reach ubRMSE ≈ 0.039 with R ≈ 0.80 on core validation sites; some SMOS Level-3 regional ubRMSE up to ≈0.10 where vegetation increases [165,174]
AMSR-E/AMSR2 (C/X/K- bands)	bias-corrected RMSE improvements reported; regional ubRMSE often >0.05 and can exceed 0.10	generally lower R and larger biases vs. L-band	Algorithm refinements (dynamic scattering/albedo) reduced bias-corrected RMSE by ~25–38% and increased R2 in test regions, but AMSR products still show large systematic biases over vegetated areas in several evaluations [176,179]

**Table 5:** Representative accuracy of surface temperature retrieval algorithms.

Mission	Retrieval Approach	Reported Accuracy (Typical Metric)
SMOS (L-band)	Iterative RTM inversion	Artic surface temperature: Median R = 0.60 (ERA5 shows R = 0.51 for sites with water fraction < 0.04) Median bias ~0.2 °C (between retrieved and in situ temperature) [213]
SMAP (L-band only passive)	Assimilation of TB in land surface model	The global land average RMSE versus in situ measurements of daily maximum temperature (T2mmax) is reduced by 0.04 K for LADAS compared to ADAS estimates. Regionally, the RMSE of LADAS T2mmax is improved by up to 0.3 K [214].
37 GHz simulated	Single-channel linear	Theoretical bias within 1 K for ~70% vegetated areas; precision < 2.5 K (forests) and <3.5 K (low vegetation) [202]
AMSR2	Deep dynamic neural network	Mean error ≈ 1.4 K, std ≈ 1.9 K; comparison to ground stations ≈ 1.8 K accuracy [203]
Multifrequency simulated database	Coupled LST–SM retrieval	LST accuracy ≈ 1.63 K (simulation validation) [204]
AMSR-E	Two-stage parameterized algorithm	Simulated RMSE 1.45 K; cross-validation daily accuracy ≈ 3.04 K, bi-monthly ≈ 4.43 K [207]
AMSR2	Physically based algorithm	Overall RMSE ≈ 5.42 K and bias ≈ 2.99 K (reported overestimation with respect to MODIS nighttime acquisitions) [205]
SSM/I	Pre-computed emissivity method	Bias ≈ 0.5 K vs. IR products, overall RMSE ≈ 5 K; well-controlled vegetated stations down to RMSE ≈ 2.5 K [208]
6–18 GHz simulations	Iterative RTM inversion	Surface temperature accuracy ≈ 2 °C achievable (except bare soils) [206]

**Table 6:** Representative accuracy of VOD retrieval algorithms.

Mission	Reference Validation Data	Reported Accuracy (Typical Metric)
SMOS and SMAP (L-band)	Ground-based in situ plant water, NDWI, crop model	Bias: 0.02–0.09 Np over agriculture crops [236]
SMOS (L-Band)	Destructive VWC (Corn)	R ≈ 0.80 (between satellite VOD and VWC) [237]
Satellite VOD (Multi-freq)	LFMC	R ≈ 0.70 to 0.85 (VOD vs. LFMC temporal dynamics) [238]
AMSR-E (multifrequency)	GNSS Normalized Microwave Reflection Index (NMRI)	R^2^ (≈0.73) and RMSE (36.8 days) for start-of-season [235]

**Table 7:** Representative accuracy of VOD vs. vegetation parameters relationships.

Mission	Reference Validation Data	Reported Accuracy (Typical Metric)
SMOS (L-band)	AGB and tree canopy height	R = 0.80–0.94 (AGB at global scale with saturation at ~360 Mg/h) [81,92,242] R = 0.87–0.90 (tree canopy height with saturation at ~30 m) [166,242]
AMSR (C/X/Ku-band)	AGB and tree canopy height	R lower than results with L-band for canopy height and AGB RMSD = 27.3 Mg·ha^−1^ [261]
SMOS (L-band)	GEDI and ICESat-2 RH100 and PAI	R = 0.65–0.94 (RH100 lower correlation on boreal forest, higher correlation on tropical forests) [226] R = 0.83–0.93 (PAI lower correlation on temperate forest, higher correlation on tropical forests; boreal forest not covered) [227]
SMOS (L-band)	Ecosystem functional properties	R^2^ ~ 0.93 (Africa), R^2^ ~ 0.87 (S. America) [249]
AMSR-2 (C/X/Ku-band)	fAPAR (Absorbed PAR)	R = 0.57 (short vegetation), 0.49 (broadleaf) [238]
AMSR-2 (C/X/Ku-band)	Leaf Area Index (LAI)	R = 0.49–0.57 [237,238]
AMSR (X-band)	VWC	R ≈ 0.43 (over grassland and crops) [262]

**Table 8:** Representative accuracy of snow and ice retrieval algorithms.

Mission	Reference Validation Data	Reported Accuracy (Typical Metric)
AMSR-2 (C/X/Ku-band)	93 meteorological stations in Northeast China	R = 0.39 and RMSE = 26.15 cm for snow depth at native resolution; R = 0.53 and RMSE = 7.58 cm for snow depth with ML downscaling [286]
AMSR-2 (C/X/Ku-band)	In situ site validation with RT model LUT over Greenland Ice Sheet (during spring)	RMSE = 0.43 m, bias 0.01 m and R = 0.89 for snow depth [288]
SMOS (L-band)	Operation IceBridge airborne (during spring 2012) over the Arctic	RMSE = 5.5 cm (up to 35 cm snow), bias = 0.1 cm (mean difference) and R^2^ = 0.58 for snow thickness [284]
SMOS (L-band)	43 ground stations over Quebec (Canada)	RMSE = 8.3 kg/m^3^, bias = 9.4 kg/m^3^ for Snow density [290]

**Table 9:** Representative accuracy of surface freeze–thaw state retrieval algorithms.

Mission	Reference Validation Data	Reported Accuracy (Typical Metric)
SSM/I (37 GHz V-pol)	Global validation vs. in situ data, multi-decadal (20 years)	classification accuracy = 92.2 ± 0.8% (PM/evening) classification accuracy = 85.0 ± 0.7% (AM/moring) [231]
SMAP (L-band)	Global validation vs. in situ data	Global mean annual classification accuracy ~78% (descending/AM) Global mean annual classification accuracy ~90% (ascending/PM) [233]
SMOS (L-band)	Validation vs. in situ data over Canada	Classification accuracy = ~87.8% [234]
AMSR-E/AMSR-2 (C/X/Ku-band)	Validation vs. in situ data over the Tibetan Plateau	Overall accuracy = ~82.0% [235]
AMSR-2 + SMAP (L/C/X/Ku-band)	Comparison vs. ERA5 over the Northern Hemisphere	Mean Percent Accuracy = 92.7% [236]

**Table 10:** Representative accuracy of flood monitoring algorithms.

Mission	Reference Validation Data	Reported Accuracy (Typical Metric)
AMSR-2 (C/X/Ku-band)	Optical flood maps (MODIS 500 m), in situ discharge, modelled inundation fractions	65–95% agreement; mean bias ≈ −0.04 ± 0.28 (flood fraction vs. MODIS, over Bangladesh) [347]
SMOS (L-band)	River gauge water levels (Amazon, Orinoco, Congo); GloFAS model assimilation	R = 0.8–0.94 (water level correlation) [348,349]
SMAP (L-band)	In situ flood extent maps, optical water indices	Producer accuracy up to ~85–90% for flood mapping (threshold ≈ 0.04 m^3^/m^3^) [350]

**Table 11:** Comparative summary of representative data assimilation methodologies for passive microwave remote sensing.

Method	Key Characteristics	Representative Applications	Strengths	Limitations	Computational Cost	Representative Performance
EnKF/LETKF	Ensemble-based, flow-dependent covariance	SMAP Tb assimilation in GEOS-5; SMOS in AWRA-L [351,353,356,357]	Captures dynamic error structures; operational maturity	Gaussian assumptions; ensemble size sensitivity	Moderate–High	RMSE ≈ 0.034 m^3^·m^−3^; correlation +0.23 gain
EKF	Linearized update around state estimate	SMOS Tb in ECMWF H-TESSEL [358]	Computational efficiency; operational simplicity	Linearization errors in nonlinear regimes	Moderate	Improved soil moisture in data-sparse regions
Particle Filter/Smoother	Sequential Monte Carlo; non-Gaussian posterior	AMSR-2 Tb → Crocus SWE; RT parameter estimation [356,359,360]	Handles strong nonlinearities; joint state/parameter estimation	High computational demand; particle degeneracy	Very High	SWE bias ↓ 68%; error ↓ 5% (relative)
Hybrid/Dual-Cycle	Joint state-bias estimation; adaptive correction	SMAP Tb bias correction [352]	Dynamic bias adaptation; improved consistency	Complex implementation; tuning requirements	Moderate–High	Enhanced retrieval stability
Machine Learning–Integrated DA	ML for downscaling, operator emulation, fusion	SMAP–SMOS–AMSR-2 merged DA [361,362]	Improves resolution; accelerates forward modeling	Requires large training datasets; generalization risk	Low–Moderate	RMSE < 0.06 m^3^·m^−3^ (high-resolution SM)

The ↓ symbol indicates a decrease in metrics with respect to the state of the art. Note that the improvement with respect to the state of the art is dependent on the specific metric, for instance ↓ related to an error like RMSE is considered an improvement with respect to other approaches in literature.

**Table 12:** Summary of major Earth system applications of passive microwave data assimilation.

Institution	Model	Assimilated Satellite Data	DA Method	Variables Updated	Key Performance Gains
NASA/GMAO	GEOS-5 Catchment LSM (L4_SM) [351,352,357]	SMAP, SMOS Tb	EnKF	Surface and root-zone soil moisture	Corr. +0.26; RMSE ↓ 0.008 m^3^·m^−3^
ECMWF	H-TESSEL [359]	SMOS Tb	EKF	Soil moisture, surface fluxes	Improved soil moisture and surface flux realism
NOAA/NASA	LIS (Noah, Noah-MP, VIC) [363]	SMOS, SMAP retrievals	EnKF	Soil moisture	Hydrological forecast accuracy ↑
BoM (Australia)	AWRA-L [358,364]	SMAP, SMOS Tb	EnKF	Soil moisture, water balance	Improved drought/flood prediction
CMA (China)	CLDAS [354]	SSM/I, AMSR-2 Tb	EnKF	Soil moisture, freeze/thaw	Enhanced regional soil moisture consistency
Météo-France	Crocus [356]	AMSR-2 *T*b	Particle Filter	SWE	SWE bias ↓ 68%; RPE ↓ 19→14.1%
AWI	AWI Climate Model [365]	AMSR-2, SSM/I Tb	Coupled DA	Sea ice concentration/thickness	Improved sea ice prediction skill
NASA	GEOS-LADAS [363]	GEOS-5 coupled model	Weakly coupled DA	Soil moisture, T2M (Temperature at 2 m above the ground), Q2m (Specific humidity at 2 m above the ground), heat fluxes	RMSE ↓ 0.04 K (T2M var.), ↓ 0.05 g·kg^−1^ (Q2M var.)

The ↑ symbol indicates an increase in metrics with respect to the state of the art. The ↓ symbol indicates a decrease in metrics with respect to the state of the art. Note that the improvement with respect to the state of the art is dependent on the specific metric, for instance ↓ related to an error like RMSE is considered an improvement with respect to other approaches in literature.

## Data Availability

No new data were created or analyzed in this study.
